# Complete Chloroplast Genome of *Enkianthus* Lour. (Ericaceae): Comparative Analysis, Phylogenetic Relationships, Divergence History, and Adaptive Evolution

**DOI:** 10.1002/ece3.72129

**Published:** 2025-09-19

**Authors:** Shuilian Peng, Zhijun Zhai, Wan Hu, Hua Liang, Yi Yang, Yixuan Kou, Meixia Wang, Shanmei Cheng, Zhiyong Zhang, Dengmei Fan

**Affiliations:** ^1^ Laboratory of Subtropical Biodiversity, College of Forestry Jiangxi Agricultural University Nanchang China; ^2^ College of Agriculture Jiangxi Agricultural University Nanchang China; ^3^ Bioengineering and Technological Research Centre for Edible and Medicinal Fungi, College of Bioscience and Bioengineering Jiangxi Agricultural University Nanchang China; ^4^ Lushan Botanical Garden, Jiangxi Province and Chinese Academy of Sciences Jiujiang China; ^5^ Key Laboratory of Ecology of Rare and Endangered Species and Environmental Protection (Guangxi Normal University), Ministry of Education Guilin China; ^6^ Jiangxi Provincial Key Laboratory of Conservation Biology College of Forestry, Jiangxi Agricultural University Nanchang China

**Keywords:** adaptive evolution, divergence time, *Enkianthus*, phylogenetic relationships, plastomes

## Abstract

*Enkianthus* (Ericaceae) is a small genus of great ornamental and ecological importance, preferring the specialized habitats in montane elfin forests of east Asia. Here, we for the first time sequenced, assembled, and annotated the complete chloroplast genomes of 16 species, representing all four sections of *Enkianthus*. The plastomes exhibited a typical quadripartite structure and were highly conserved in genome size, organization, and gene content. Plastid phylogenomics confirmed the monophyly of four sections of *Andromedina*, *Enkiantella*, *Enkianthus*, and *Meisteria*, with sect. *Enkianthus* as the first diverging clade. Divergence time analysis supported an ancient origin of *Enkianthus* at the Late Cretaceous but rapid recent radiation since the Lat‐Miocene onward. Selection pressure analysis detected 12 positively selected genes, which might be linked to adaptive evolution in *Enkianthus*. These results will provide invaluable information for further population‐level studies on species delimitation and adaptive evolution in *Enkianthus*, and will benefit *Enkianthus* conservation and utilization.

## Graphical abstract

The *Enkianthus* plastomes were first reported and compared. *Enkianthus* originated in the Late Cretaceous but diverged after the Lat‐Miocene. Genome‐wide scanning identified 12 positive selection genes relevant to adaptive evolution.

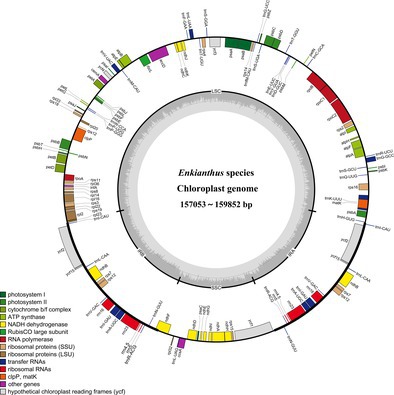

## 1 Introduction

Chloroplasts are organelles evolved gradually from a cyanobacterium engulfed by a eukaryotic cell (Martin and Kowallik 1999). Each harbors its own genome that is typical maternal inheritance (with rare paternal or biparental inheritance) (Daniell et al. 2021). During the long evolutionary process, the majority of endosymbiont genes were transferred to the host nuclear genome, with only about 100 genes remaining in the chloroplasts (Jarvis and López‐Juez 2013). The plastid genome has been extensively used to resolve the deep phylogeny and evolution of plants due to its uniparental inheritance, low rate of nucleotide evolution, lack of recombination, and conservation of genome content and structure (Daniell et al. 2021), but with sufficient characters for phylogenetic reconstruction. In addition, comparative analyses of plastomes among closely related plant species could provide in‐depth insights into patterns of evolutionary dynamics and micro‐structural genome organization, resulting in deeper knowledge about the micro‐evolution of chloroplast genes and genomes in flowering plants (Gao et al. 2019).

Ericaceae comprises ca. 4000 extant species across 125 genera, making it a species‐rich family of woody plants distributed globally (Fang et al. 2005). Most members of this family are vital components of montane ecosystems, and many are renowned for their horticultural value. Consequently, an increasing number of whole plastid genomes have been sequenced to study molecular systematics and adaptive evolution in this family, particularly within the hyper‐diverse genus *Rhododendron* (Mo et al. 2022; Xia et al. 2022). *Enkianthus* Lour., a small genus within Ericaceae, consists of about 12–17 species mainly distributed in subtropical China (including Taiwan) and Japan (Anderberg 1994; Fang and Stevens 2005; Kron et al. 2002). Most of these species possess significant economic and ornamental value, characterized by their elegant tree crowns and distinctive bell‐shaped corolla. They serve as excellent fresh‐cut branches for floral arrangements. Several have already been introduced into horticultural cultivation in gardens across Japan and China. Based on morphology, anatomy, embryology, and cytology, Anderberg (1994) classified *Enkianthus* into four sections, that is, *Enkianthus*, *Andromedina*, *Meisteria*, and *Enkiantella*. Each of the former two sections was supported as a monophyletic clade in the molecular phylogenetic study of Tsutsumi and Hirayama (2012) based on combined sequence datasets of *ITS*, *matK*, *trnK* intron, and *trnS*‐*trnG* spacer, but the monophyly of the latter two sections remained unresolved due to insufficient variation in chloroplast DNA (cpDNA) markers. Recently, we reconstructed the phylogeny of *Enkianthus* using six cpDNA regions and two nuclear loci, with a special attempt to decipher the origin of polyploids within the genus (Zhou et al. 2024). All four sections were confirmed as monophyletic in the cpDNA phylogenetic tree and suggested the robustness of molecular analysis could be increased with the increasing number of loci. However, to date, no whole chloroplast genome of the genus *Enkianthus* has been reported, which is now fairly convenient to obtain via high‐throughput data collection and low cost of next‐generation DNA sequencing (Shendure and Ji 2008). Comparative analysis of plastomes may provide a more accurate and rapid strategy to differentiate closely related taxa in contrast with the use of limited chloroplast regions (Li et al. 2014). Furthermore, natural hybridization and polyploidization are frequent in Ericaceae plants and play a crucial role in plant evolution and speciation (Mo et al. 2022; Zheng et al. 2021). Although *Enkianthus* is species‐poor, polyploidy and suspected hybrid origins have been reported in this genus (Liang et al. 2022; Zhou et al. 2024). Plastid genomes are particularly useful for tracing maternal ancestry and will provide a comprehensive picture of these processes when combined with nuclear data (e.g., low‐copy nuclear genes, transcriptomes, or whole‐genome sequencing). Additionally, a well‐resolved phylogenetic tree would allow us to estimate the timing and history of diversification in *Enkianthus*. Previous estimates of Ericaceae's origin vary, ranging from ~90 Mya (24 plastid genes + *ITS*; Rose et al. 2018) to ~117.28 Mya (*rbcL* + *matK*; Schwery et al. 2015). Given that *Enkianthus* is the earliest‐diverging lineage in Ericaceae (Kron and Judd 1997; Kron et al. 2002), resolving its divergence time would greatly advance our understanding of the family's evolutionary origin.

This genus also holds significant ecological importance, as several of its species (e.g., *E. quinqueflorus*, *E. serrulatus* and *E. chinensis* ) serve as dominant components of subtropical montane elfin forests in east Asia (Schwery et al. 2015). The montane elfin forest trees normally grow at isolated summit locations or mountain ridges, facing extreme environmental conditions, that is, strong winds, lower temperatures, and poor soils (Yao et al. 2017). Such severe environmental conditions likely impose *intense* selective pressures, potentially leaving detectable signatures in genes involved in adaptive evolution (Li, Zheng, et al. 2024; Li, Nie, et al. 2024; Xia et al. 2024). The chloroplast is an essential organelle in plant cells and is responsible for various crucial functions, including photosynthesis, lipid metabolism, and amino acid biosynthesis (Jensen and Leister 2014). As such, an increasing number of studies have detected certain chloroplast genes under positive selection and traced adaptive changes across taxa (Huang et al. 2024). For instance, Zhang et al. (2022) detected three chloroplast genes (*clpP*, *rbcL*, and *ccsA*) under positive selection in *Ficus*, potentially facilitating adaptation to diverse habitats. Li, Zheng, et al. (2024) and Li, Nie, et al. (2024) identified seven positively selected genes linked to the adaptation of *Firmiana danxiaensis* to Danxia and Karst landforms. Thus, the chloroplast genomes represent a good system to trace adaptative genetic architecture through evolutionary history. A plastid‐level genomic analysis of *Enkianthus* could provide novel insights into the ecological adaptation of its representatives in extreme montane environments.

In this study, we conducted chloroplast (cp) genome sequencing and assembly of 16 species of *Enkianthus*, representing all four recognized sections of the genus (Table S1). Based on whole‐plastome sequences, phylogenomic analysis was performed to clarify evolutionary relationships within the genus toward obtaining an in‐depth view of chloroplast gene and genome evolution. Our specific goals were as follows: (1) to compare the plastid genomes and identify structural variants in *Enkianthus* and (or) across Ericaceae; (2) to infer and test the phylogenetic relationships among the species and sections using plastome data; (3) to estimate the timing and history of diversification in *Enkianthus*; (4) to scan for positively selected protein‐coding genes, which in particular are expected to play an important role in adaptations to their ecological niches in east Asia.

## 2 Materials and Methods

### 2.1 Sampling, Extraction and Genome Sequencing

We sampled a total of 16 species within the genus *Enkianthus*, including some taxa that have been treated as synonyms (e.g., *E. calophyllus*, *E. tubulatus*) in Flora of China (Fang and Stevens 2005). Detailed information of the sampled species is provided in Table S1. Fresh leaf material was collected, dried using silica gel, and then stored at −20°C. Chloroplast DNA was isolated from dried leaves using a modified high salt method (Dellaporta et al. 1983). Eleven of the sampled species were then sent for complete chloroplast sequencing. The Illumina TruSeq Library Preparation Kits were used to construct an Illumina paired‐end DNA library. The library was sequenced on the Illumina Hiseq 2500 sequencing platform, and then sequence reads of 2 × 150 bp were obtained. For the remaining five species (*E*. *calophyllus*, *E. quinqueflorus* var. *ciliatoserrulatus*, *E. tubulatus*, *E. chinensis* , and *E. cernuus* f. *rubens*), our unpublished whole‐genome re‐sequencing data were used for the following chloroplast genome sequence assembly.

### 2.2 Genome Assembly and Annotation

The raw data were filtered by Fastp v. 0.22.0 (Chen et al. 2018), and then *de novo* assembly was performed on the clean data using GetOrganelle v. 1.7.7.0 (Jin et al. 2020) with default parameters. Two reference cp genomes served as initial seeds for read recruitment: *Clethra fargesii* (Genbank No.: MT742578) and *Vaccinium bracteatum* (Genbank No.: LC521967). To check the accuracy of sequence assembly, we used Bowtie v. 2.5.1 (Langmead and Salzberg 2012) to match high‐quality data to the assembled sequences, and then visualized them using IGV v. 2.11.9 (Robinson et al. 2011) to observe base coverage. The depth profile was generated via Perl scripts.

The assembled cp genomes were annotated by PGA (Plastid Genome Annotator, https://github.com/quxiaojian/PGA) (Qu et al. 2019), also using the above cp genomes of *C. fargesii* and *V. bracteatum* as the reference genomes. The software Geneious v. 9.0.2 (Kearse et al. 2012) was used to visualize the annotation results and adjust the errors manually. Finally, the circular cp genomic maps of *Enkianthus* species were drawn and visualized by OGDRAW (OrganellarGenomeDRAW, https://chlorobox.mpimp‐golm.mpg.de/OGDraw) (Lohse et al. 2007). The newly assembled and annotated sequences were submitted to GenBank (Table 1).

**TABLE 1:** Summary of chloroplast genome features of the 16 studied *Enkianthus* species.

Species	GenBank accession no.	Length (base pair, bp)				Number of genes					GC content (%)			
		Complete	LSC	SSC	IR	Complete	PCGs	tRNA	rRNA	Intron‐containing	Complete	LSC	SSC	IR
*E. calophyllus*	PP737346	157,053	89,561	20,330	23,581	114	80	30	4	19	37.68	36.02	31.65	43.45
*E. perulatus*	PP727293	157,240	89,670	20,450	23,560	114	80	30	4	19	37.69	36.02	31.59	43.49
*E. quinqueflorus* var. *ciliatoserrulatus*	PP737348	158,683	90,835	20,542	23,653	114	80	30	4	19	37.68	36.04	31.64	43.46
*E. quinqueflorus*	PP727290	158,689	90,854	20,377	23,729	114	80	30	4	19	37.68	36.05	31.74	43.37
*E. serrulatus*	PP727288	159,277	91,202	20,551	23,762	114	80	30	4	19	37.70	36.09	31.68	43.41
*E. tubulatus*	PP727297	159,300	91,219	20,557	23,762	114	80	30	4	19	37.67	36.04	31.66	43.41
*E. serotinus*	PP727294	158,992	90,900	20,636	23,728	114	80	30	4	19	37.68	36.05	31.61	43.43
*E. nudipes*	PP727296	158,300	89,634	18,618	25,024	114	80	30	4	19	37.74	36.08	32.23	42.78
*E. subsessilis*	PP727295	158,288	89,403	18,679	25,103	114	80	30	4	19	37.71	36.07	32.14	42.72
*E. ruber*	PP737349	159,852	91,190	19,968	24,347	114	80	30	4	19	37.69	36.08	31.98	43.05
*E. chinensis*	PP727289	159,697	91,024	19,863	24,405	114	80	30	4	19	37.67	36.05	31.99	43.00
*E. deflexus*	PP727292	159,586	90,875	20,071	24,320	114	80	30	4	19	37.68	36.08	31.85	43.06
*E. campanulatus*	PP722976	158,033	89,928	19,853	24,126	114	80	30	4	19	37.66	36.02	31.86	43.12
*E. cernuus* f. *rubens*	PP737347	159,558	90,922	19,826	24,405	114	80	30	4	19	37.68	36.05	31.95	43.05
*E. sikokianus*	PP737350	159,197	90,714	19,767	24,358	114	80	30	4	19	37.72	36.10	32.00	43.08
*E. cernuus*	PP727291	159,575	91,128	19,801	24,323	114	80	30	4	19	37.69	36.05	31.93	43.08
Mean	—	158,831	90,566	19,993	24,137	114	80	30	4	19	37.69	36.06	31.84	43.19

Abbreviations: GC, guanine‐cytosine; IRs, inverted regions; LSC, large single‐copy; PCGs, protein‐coding genes; SSC, small single‐copy region.

### 2.3 Genome Structure Analyses

The software Geneious v. 9.0.2 was also employed to calculate the basic structural characteristics of plastomes, such as genome length, GC content, and gene number. The simple sequence repeats (SSRs) were screened by MISA (MicroSAtellite identification tool, https://webblast.ipk‐gatersleben.de/misa/) (Beier et al. 2017). Following Li et al. (2021), the minimum repeat thresholds in the analysis were set as eight for mononucleotides, five for dinucleotides, and three for tri‐ through hexanucleotides. The complex repeat sequences were classified into three categories: tandem, palindromic, and dispersed repeats. We determined tandem repeats using Tandem Repeats Finder v. 4.09 (Benson 1999) with the parameters “2 7 7 80 10 50 500 –f –d –m.” Vmatch v. 2.3.1 (Kurtz 2010) was used to search palindromic (≥ 30 bp) and dispersed repeats (≥ 30 bp). We investigated the codon usage of all the coding sequences (CDSs) and performed the codon bias analysis. Relative synonymous codon usage (RSCU) and the codon numbers were calculated by CodonW v. 1.4.2 (Meade et al. 1997).

### 2.4 Comparative Analysis of the Chloroplast Genome

The newly assembled plastomes of *Enkianthus* were compared by the online program VSTA (https://genome.lbl.gov/vista/mvista/submit.shtml) (Frazer et al. 2004) with the Shuffle‐LAGAN model, with default parameters and using the annotated chloroplast genome of *Enkianthus perulatus* as a reference genome. CPJSdraw v. 0.01 was used to compare the boundary genes between the inverted repeat (IR) and single copy (SC) regions of each plastome (Li et al. 2023). We calculated nucleotide diversity (Pi) to identify highly variable regions among the plastomes by DnaSP v. 6.12.03 (Rozas et al. 2017) with a step size of 200 bp and a window length of 600 bp.

### 2.5 Phylogenetic Analysis

To elucidate the phylogeny of *Enkianthus*, we respectively constructed phylogenetic trees based on the complete chloroplast genome sequences (WG) and 78 protein coding genes (PCGs) of the 16 *Enkianthus* species and two outgroups ( *C. fargesii* and *V. bracteatum* ). The sequences were aligned using the software MAFFT v. 7.313 (Katoh and Standley 2013), and concatenated by PhyloSuite v. 1.2.2. Subsequently, the phylogenetic relationships were inferred by Maximum Likelihood (ML) and Bayesian Inference (BI) methods. ML trees were constructed by RAxML v. 8.2.12 (Stamatakis 2014) with the GTRGAMMA model and 1000 bootstrap replications. For BI analyses, the best‐fitted models for the WG and PCGs datasets were TVM + G and GTR + I + G, respectively, which were selected according to the Akaike information criterion (AIC) implemented in jModelTest v. 2.1.7 (Darriba et al. 2012). BI trees were constructed (2,000,000 generations, sampling every 1000 generations) using MrBayes v. 3.2.7 (Ronquist et al. 2012), with the initial 25% of sampled data discarded as burn‐in. Convergence was confirmed by split frequencies < 0.01 and ESS > 200.

### 2.6 Divergence Time Estimation

To estimate the divergence times of the *Enkianthus* lineages, we used the WG matrix of the 16 newly assembled sequences and eight other chloroplast genome sequences downloaded from GenBank (Table S2). We followed the method of Ma et al. (2021) using the fossils of crown Ericales (89 Ma) and stem *Rhododendron* (56 Ma) as two calibration points. A Bayesian dating method was employed to infer divergence time using the program BEAST v. 1.5.4 (Drummond and Rambaut 2007), with the concatenated alignment treated as a single locus. The analysis used an unlinked GTR model of nucleotide substitution and gamma‐distributed rate variation. A relaxed molecular clock using the uncorrelated log‐normal model was applied with a Yule process speciation prior for branching rates. The Markov chain Monte Carlo (MCMC) analysis was run for 40 million generations (burn‐in 10%) with parameters sampled every 1000 steps to ensure effective sample size (ESS) values above 200 for most parameters in Tracer v. 1.7.2 (Rambaut et al. 2018). The final maximum clade credibility tree with median node ages and 95% highest posterior density (HPD) intervals was visualized using FigTree v. 1.3.1.

### 2.7 Positive Selection Analysis

We scanned all 80 PCGs of *Enkianthus* to identify positively selected chloroplast genes using PAML v. 4.9 (Yang 2007). The analysis was performed with the genome‐wide phylogenetic ML tree (excluding outgroups). We calculated the non‐synonymous substitution rate (*dN*), synonymous substitution rate (*dS*), and their ratio (*ω* = *dN*/*dS*). We applied site (M1a & M2a) and branch site models (MA null [fix_omega = 1 and omega = 1] & modified MA) to test 80 PCGs with the widely employed codon frequency model (F3 × 4). The designation of foreground branches in the branch‐site model is shown in Figure S1. To evaluate the strength of selection, a likelihood ratio test (LRT) was conducted by the comparison (M1a vs. M2a; MA null vs. modified MA) against a chi‐square distribution. The positively selected genes at a significance level of *p* < 0.05 were shown. Additionally, amino acid sites under positive selection were identified by Bayes Empirical Bayes (BEB) (Yang 2005) approach, using a posterior probability greater than 0.95 as a threshold.

## 3 Results

### 3.1 Genome Size, Gene Content and Structure of *Enkianthus* Chloroplast

**FIGURE 1:**
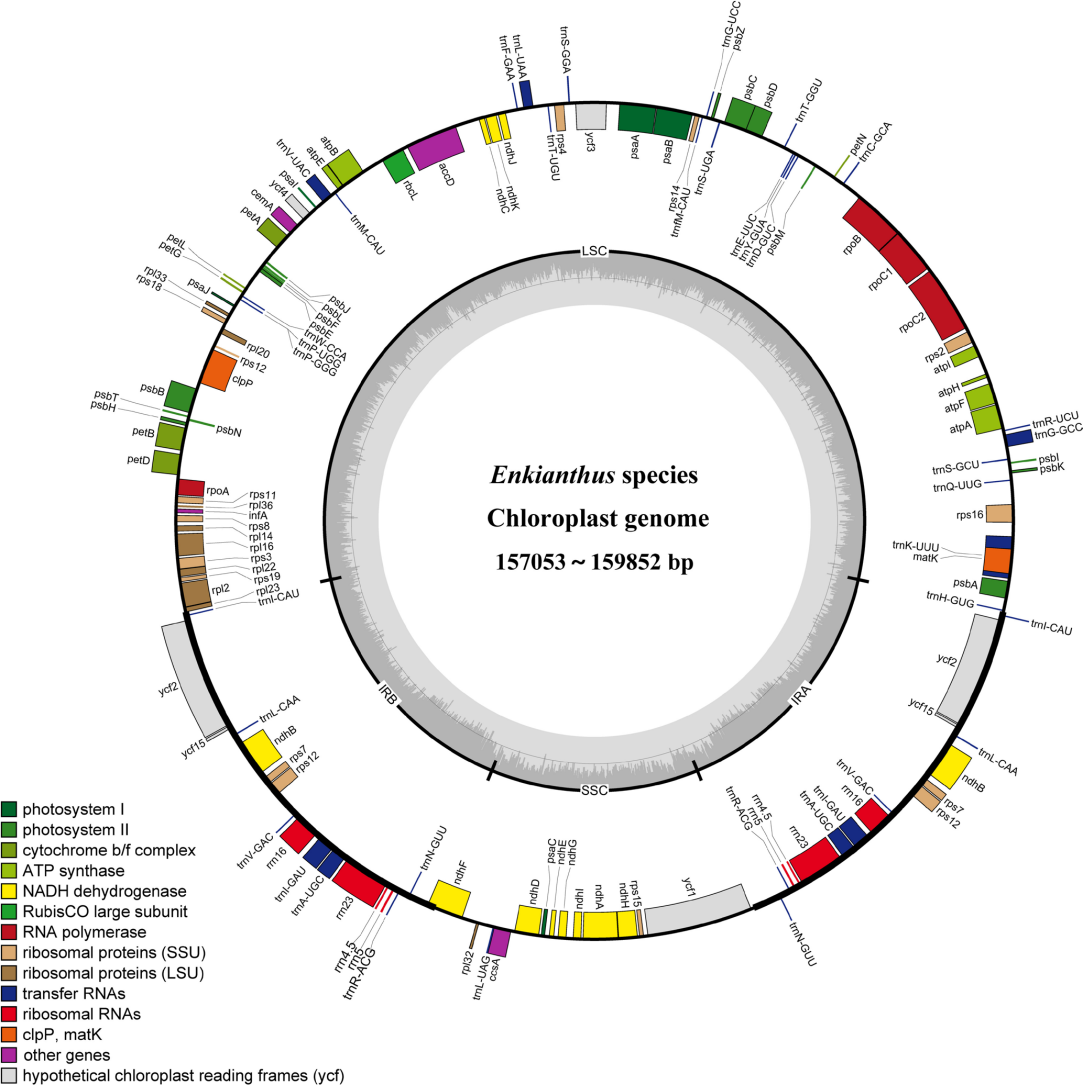
Gene map of *Enkianthus* plastomes. The inner circle shows quadripartite structure of the genome, including the large and small single‐copy regions (LSC, SSC) and the two inverted repeat regions (IRa, IRb). Genes inside the circle are transcribed clockwise, genes outside of the circle are transcribed counterclockwise. Genes belonging to different functional groups are marked in different colors. The darker gray area in the inner circle corresponds to GC content, while the lighter gray area corresponds to AT content.

The lengths of the chloroplast genomes of *Enkianthus* varied slightly, ranging from 157,053 bp (*E*. *calophyllus*) to 159,852 bp ( *E. ruber* ) (Table 1). The plastomes exhibited a typical circular quadripartite structure, consisting of two inverted repeat (IR) regions (23,560–25,103 bp) separated by a large single‐copy (LSC) region (89,403–91,219 bp) and a small single‐copy (SSC) region (18,618–20,636 bp) (Figure 1). They contained a similar total GC content of ~37.7%, and the GC content of the single‐copy region (LSC: ~36% and SSC: ~32%) was lower than that of the IR region (~43%).

The plastomes of *Enkianthus* displayed conservation in gene content with a total of 114 genes identified, including 80 protein coding genes (PCGs), 30 tRNA genes, and four rRNA genes (Table 1). Furthermore, introns were found within 16 genes, among which three genes (*clpP*, *rps12*, and *ycf3*) each had two introns, and the remaining 13 genes each contained one intron (Table 2). A trans‐spliced gene *rps12* was annotated, with one 5′_end exon located in the LSC region and two 3′_end exons situated within IRs.

**TABLE 2:** Functional annotation of chloroplast genes in the *Enkianthus* chloroplast genomes.

Categories	Gene annotation	Name of genes
Self replication	Large subunit of ribosome	rpl2*, *rpl14*, rpl16*, *rpl20*, *rpl22*, *rpl23, rpl32*, *rpl33*, *rpl36*
	Small subunit of ribosome	*rps2*, *rps3*, *rps4*, *rps7*, *rps8*, *rps11*, rps12**, *rps14*, *rps15*, *rps16*, *rps18*, *rps19*
	RNA polymerase	*rpoA*, *rpoB*, rpoC1*, *rpoC2*
	rRNA genes	*rrn4.5*, *rrn5*, *rrn16*, *rrn23*
	tRNA genes	trnA‐UGC*, *trnC‐GCA*, *trnD‐GUC, trnE‐UUC*, *trnF‐GAA*, *trnfM‐CAU*, *trnG‐GCC*, *trnG‐UCC*, *trnH‐GUG*, *trnI‐CAU*, trnI‐GAU*, trnK‐UUU*, *trnL‐CAA*, trnL‐UAA*, *trnL‐UAG*, *trnM‐CAU*, *trnN‐GUU*, *trnP‐UGG*, *trnQ‐UUG*, *trnR‐ACG*, *trnR‐UCU*, *trnS‐GCU*, *trnS‐GGA*, *trnS‐UGA*, *trnT‐GGU*, *trnT‐UGU*, *trnV‐GAC*, trnV‐UAC*, *trnW‐CCA*, *trnY‐GUA*
Photosynthesis	Subunits of photosystem I	*psaA*, *psaB*, *psaC*, *psaI*, *psaJ*, ycf3**, *ycf4*
	Subunits of photosystem II	*psbA*, *psbB*, *psbC*, *psbD*, *psbE*, *psbF*, *psbH*, *psbI*, *psbJ*, *psbK*, *psbL*, *psbM*, *psbN*, *psbT*, *psbZ*
	Subunits of NADH‐dehydrogenase	ndhA*, ndhB*, *ndhC*, *ndhD*, *ndhE*, *ndhF*, *ndhG*, *ndhH*, *ndhI*, *ndhJ*, *ndhK*
	Subunitsof cytochrome b/f complex	*petA*, petB*, petD*, *petG*, *petL*, *petN*
	Subunits of ATP synthase	*atpA*, *atpB*, *atpE*, atpF*, *atpH*, *atpI*
	Large subunit of rubisco	*rbcL*
Other genes	Translational initiation factor	*infA*
	Maturase	*matK*
	Protease	clpP**
	Carbon metabolism	*cemA*
	c‐type cytochrom synthesis gene	*ccsA*
	Subunit of acetyl‐coA‐carboxylase	*accD*
Unknown function	Conserved Open Reading Frames	*ycf1*, *ycf2*, *ycf15*

*Note:* One (*) and two (**) asterisks indicate one‐ and two‐introns in genes, respectively.

**FIGURE 2:**
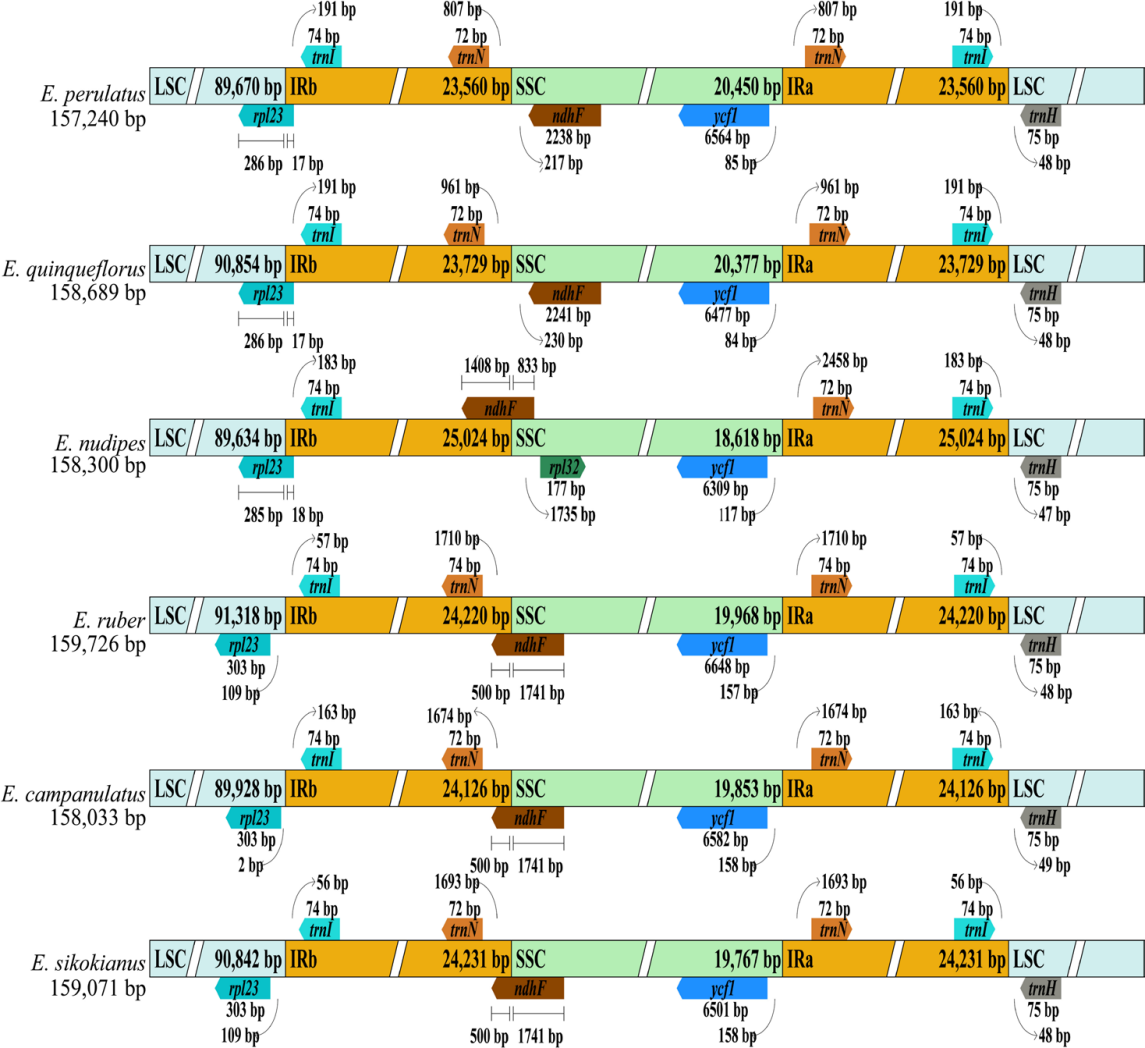
Comparison of LSC, SSC, and IR boundary positions across 16 *Enkianthus* plastomes, with six representative species shown to illustrate key differences among the four sections. Genes adjacent to or spanning junction boundaries are indicated by color‐coded arrows.

In addition, the mVISTA analysis indicated that no gene inversions or structural rearrangements were identified among the cp genomes of *Enkianthus* (Figure S2). As a whole, the plastomes demonstrated high conservation, especially the highest similarity in the IR regions. The borders of LSC/IRa and SSC/IRb included *rpl23*, *trnI‐CAU*, *trnN‐GUU*, *ndhF*, *rpl32*, *ycf1*, and *trnH‐GUG* genes. These genes at the boundaries showed slight differences among the *Enkianthus* cp genomes (Figure 2). For instance, the *rpl23* gene was found to span the boundary of LSC/IRb except that in *E. ruber* , *E. campanulatus*, and *E*. *sikokianus*. In particular, the *ndhF* and *ycf1* gene distribution varied among plastomes of the four *Enkianthus* sections. The *ndhF* gene of sect. *Enkianthus* was located in the SSC region, while the *ndhF* gene of the remaining three sections (*Andromedina*, *Meisteria* and *Enkiantella*) extended into the IRb region. The *ndhF* gene even exhibited a 1408 bp extension into the IRb region in sect. *Andromedina*. The distances of *ycf1* from the SSC/IRa junction varied from 84/85 bp in sect. *Enkianthus* to 117 bp in sect. *Andromedina* and to 157/158 bp in sect. *Meisteria* and sect. *Enkiantella* (Figure 2).

### 3.2 Repeat Sequence Analysis

**FIGURE 3:**
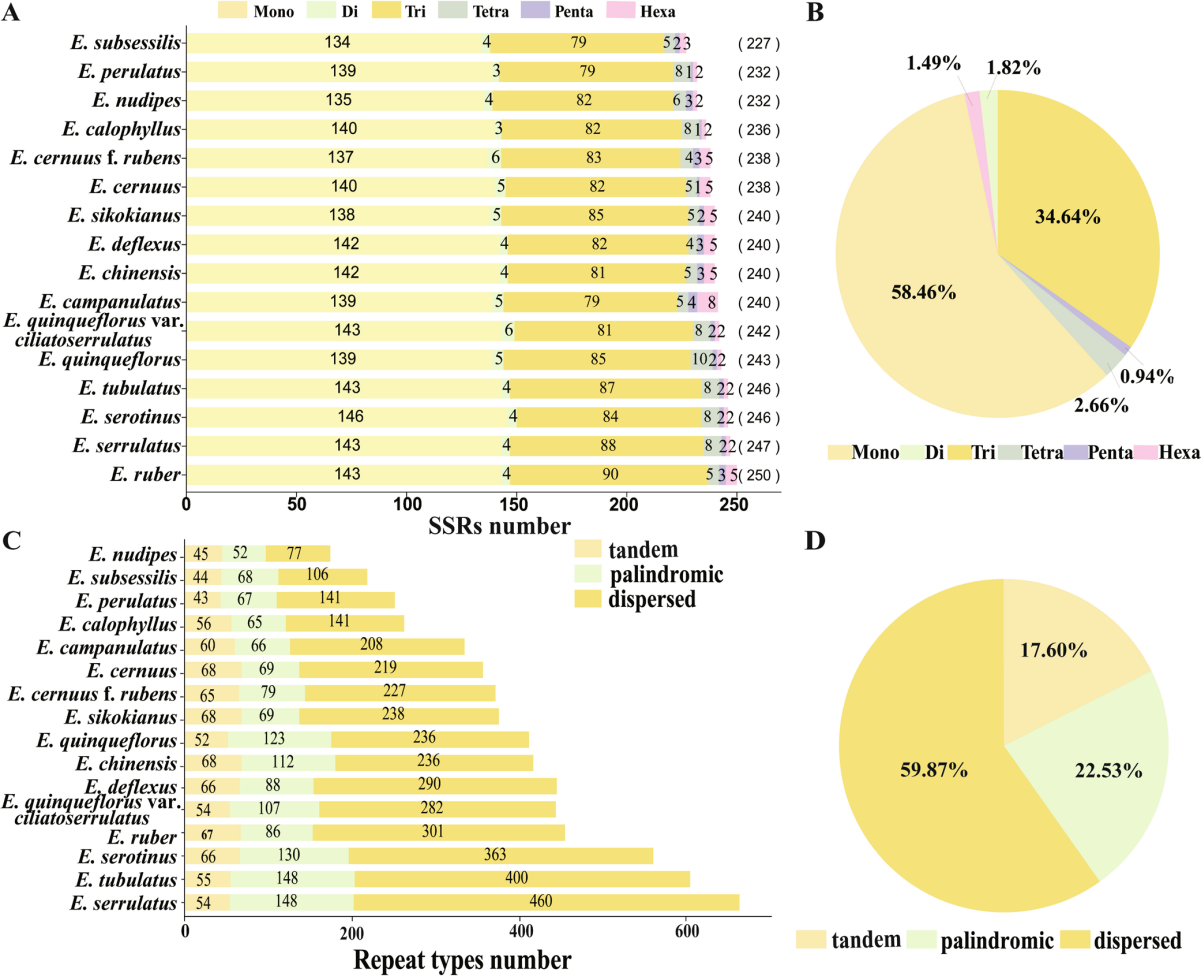
The distribution and frequency of different SSR motif types and complex repeats in 16 *Enkianthus* plastomes. (A) The number of different SSR motif types; (B) The percentage of each SSR motif type; (C) The number of different types of complicated repeats; (D) The percentage of each complicated repeat type.

The distribution and frequency of different SSR motif types (mono‐, di‐, tri‐, tetra‐, penta‐, and hexa‐nucleotide repeats) showed a high degree of collinearity among the 16 *Enkianthus* plastomes (Table S3a and Figure 3A). The mononucleotide repeats were the most abundant (58.46%), followed by trinucleotide (34.64%) and tetranucleotide (2.66%) repeats, while the least abundant SSRs were the pentanucleotide repeats (0.94%) (Figure 3B). Additionally, a total of 188–673 complicated repeats were identified in *Enkianthus* plastomes, including 58–85 tandem (Rt), 52–148 palindromic (Rp), and 77–460 dispersed (Rd) repeats (Table S3b and Figure 3C). Overall, Rd repeats were more prevalent in *Enkianthus*, accounting for 59.87% of all repeat types, followed by Rp repeats (22.53%) and Rt repeats (17.60%) (Figure 3C). Notably, we found the number of Rd. repeats varied significantly across *Enkianthus* species from 77 in *E. nudipes* to 460 in *E. serrulatus* (Table S3b and Figure 3C).

### 3.3 Nucleotide Diversity Analysis

**FIGURE 4:**
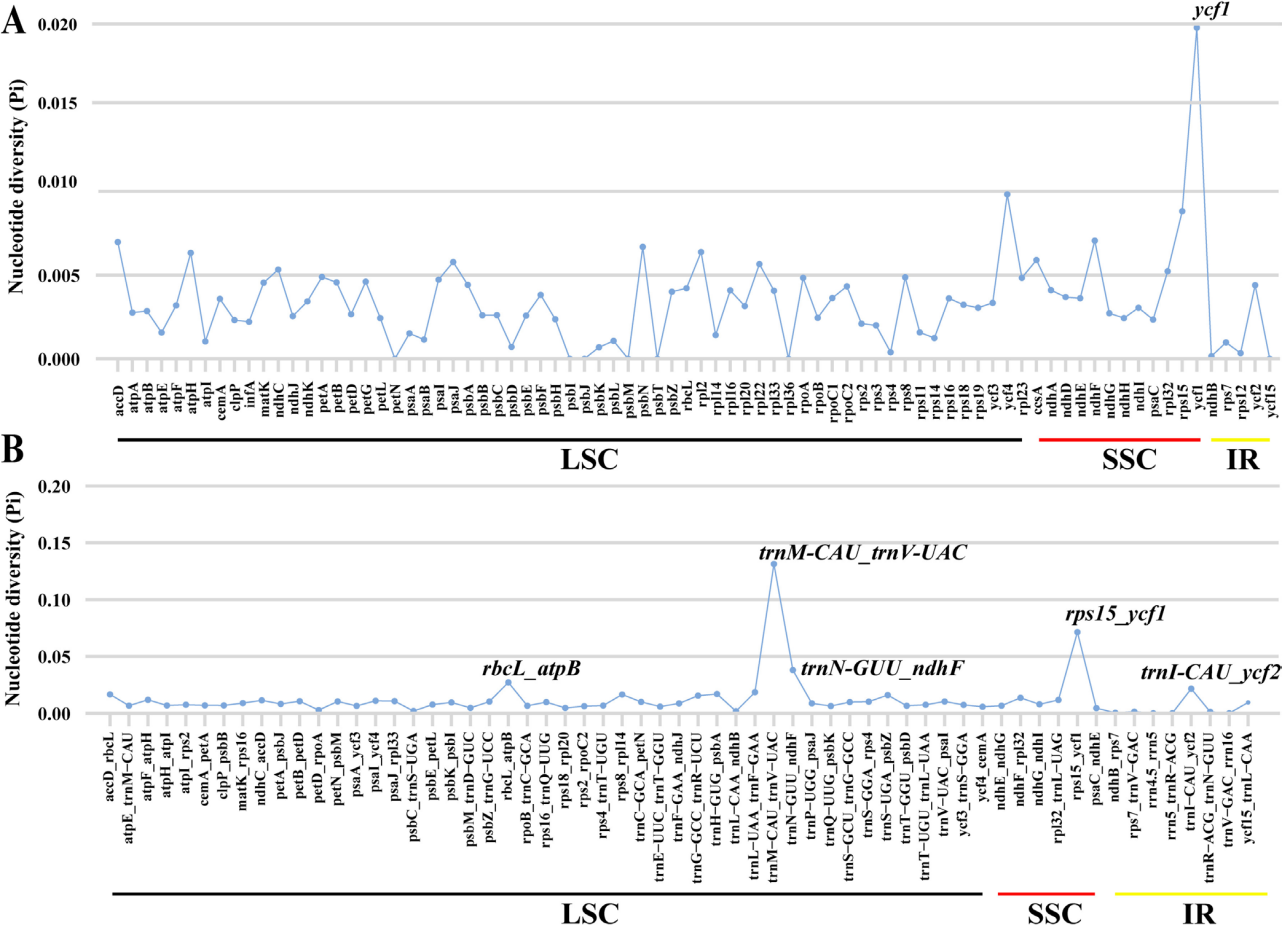
Sliding window analysis of 16 *Enkianthus* plastomes. (A) Comparison of the nucleotide diversity (Pi) among CDS regions. (B) Comparison of the nucleotide diversity (Pi) among IGS regions. The CDS and IGS names are represented by horizontal coordinates, the Pi values are represented by vertical coordinates.

In the CDS region, the nucleotide diversity (Pi) values ranged from 0 to 0.01979, with an average value of 0.00339 (Table S4a). The average Pi value was shown to be the highest (0.00573) in the SSC region, followed by the LSC region (0.00313), whereas the IR region had the lowest nucleotide diversity (0.00117). Among these regions, only the *ycf1* gene was found to harbor a Pi value higher than 0.01 (Table S4a and Figure 4A). By comparison, the intergenic spacer (IGS) regions showed considerably higher Pi values, with an average value of 0.01200 (Table S4b). The average Pi value in the LSC, SSC, and IR regions was 0.01258, 0.01930, and 0.00409, respectively, also exhibiting the highest nucleotide diversity in the SSC region and the lowest nucleotide diversity in the IR region (Table S4b and Figure 4A). In addition, 22 IGS genes were detected to be with Pi values higher than 0.01. Five hypervariable loci (Pi > 0.02) were identified in the IGS region, including *trnI‐CAU_ycf2*, *rbcL_atpB*, *trnM‐CAU_trnV‐UAC*, *trnN‐GUU_ndhF*, and *rps15_ycf* (Table S4b and Figure 4B).

### 3.4 Phylogenetic Analysis

**FIGURE 5:**
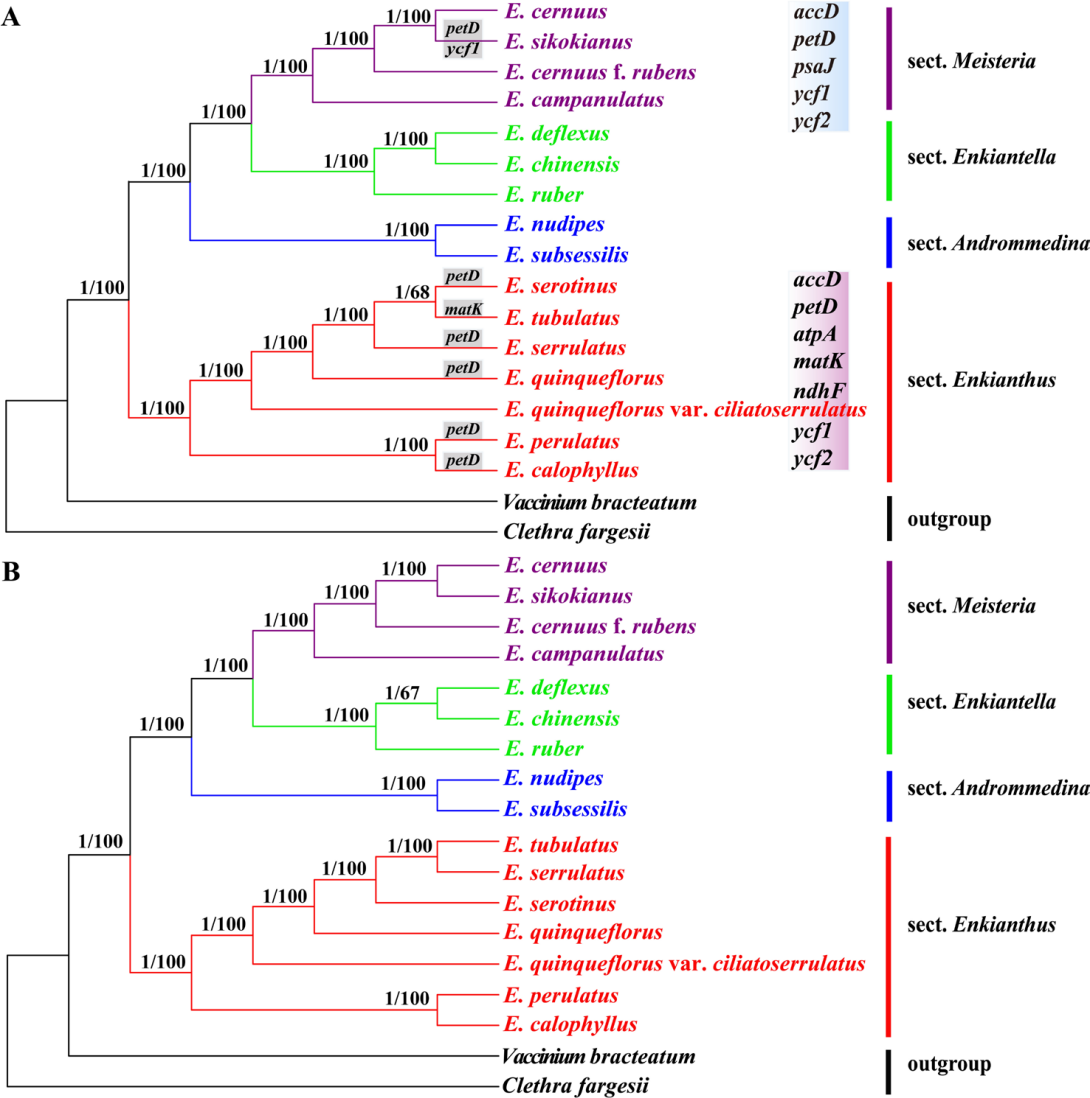
Phylogenetic trees of *Enkianthus* inferred from (A) the whole‐genome chloroplast sequences (WG) and (B) the protein‐coding genes of plastomes (CDS) using Maximum Likelihood (ML) analysis. The *Clethra fargesii* and *Vaccinium bracteatum* are used as the outgroups. PP ≥ 0.95 and BS ≥ 75% are indicated around the branches. Purple and blue rectangles respectively represent branches of sect. *Enkianthus* and sect. *Meisteria*, where genes are significantly under positive selection. Gray rectangles represent species where genes are significantly under positive selection.

The phylogenetic trees displayed similar topologies with high bootstrap support values or posterior probabilities across two datasets (WG and PCG) and two construction methods (ML and BI). The four sections—*Enkianthus*, *Enkiantella*, *Meisteria*, and *Andromedina*—were each resolved as monophyletic with strong support (Figure 5). Section *Enkianthus* was recovered as the first‐diverging group of *Enkianthus*, and the three remaining sections were clustered into a monophyletic clade. However, a slight conflict was noted in the phylogenetic placement of *E. tubulatus*, which grouped with *E. serotinus* based on the WG dataset (Figure 5A), but was sister to *E. serrulatus* in the phylogenetic tree of the PCG dataset (Figure 5B).

### 3.5 Divergence Time Estimation

**FIGURE 6:**
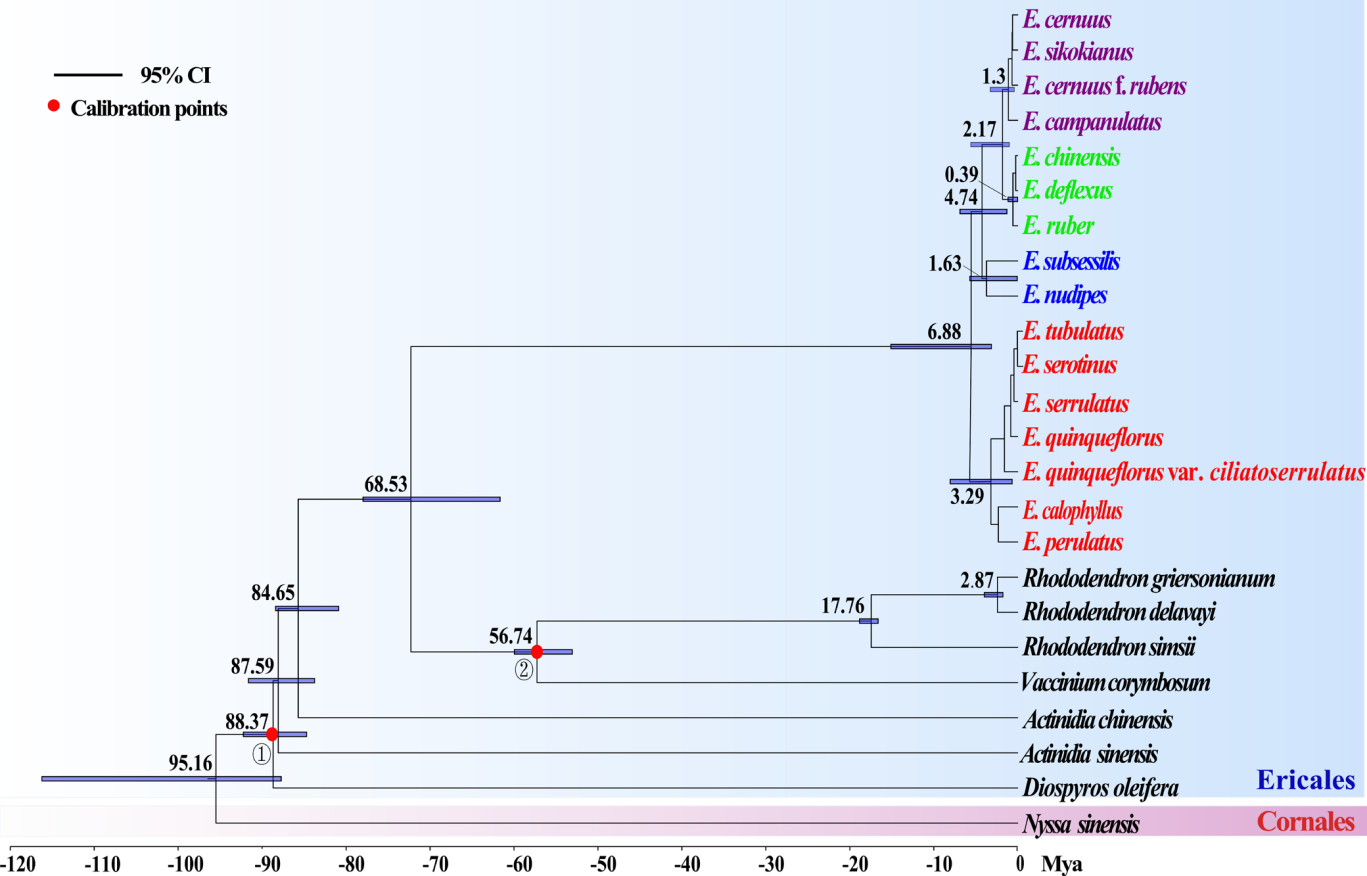
The time‐calibrated phylogenetic tree of *Enkianthus* based on whole‐genome chloroplast sequences. Nodes are numbered by estimated median divergence time using a relaxed molecular clock model with two fossil priors (red dots). The blue bars on the node represent the estimated 95% HPD intervals.

Based on sequence data from the complete chloroplast genomes, the divergence time analysis suggested that the stem age of Ericaceae was estimated at 84.65 million years ago (Mya) [95% highest posterior density (HPD): 80.88–88.39 Mya] during the Late Cretaceous (Figure 6). The differentiation time of *Enkianthus* from other genera in Ericaceae was approximately 68.53 Mya, with a 95% HPD of 61.65–77.97 Mya (Figure 6). Within the *Enkianthus* lineage, the first divergence of sect. *Enkianthus* from the other three sections arose in the Late Miocene at 6.88 Mya (95% HPD: 3.13–15.11 Mya) (Figure 6). Additionally, diversification within sections *Enkianthus*, *Enkiantella*, *Meisteria*, and *Andromedina* respectively initiated at 3.29 Mya (95% HPD: 0.66–8.04 Mya), 0.39 Mya (95% HPD: 0.06–1.21 Mya), 1.3 Mya (95% HPD: 0.41–3.28 Mya) and 1.63 Mya (95% HPD: 0.06–5.71 Mya) (Figure 6).

### 3.6 Codon Preference Analysis

The protein‐coding sequences of the 16 *Enkianthus* plastomes contained the number of codons ranging from 23,232 ( *E. nudipes* ) to 23,939 (*E. tubulatus*). The most codons (2474–2560) encoded leucine (Leu) and the fewest codons (257–261) encoded cysteine (Cys) (Table S5a and Figure S3A). After calculating the RSCU values of the 61 synonymous codons in each species, 29 or 30 codons had RSCU values greater than 1, of which 13 or 14 ended with A, 16 ended with U, and only one ended with G (UUG). This revealed a preference for A/U‐terminated codons in the chloroplast genomes. AUG (Met), UGG (Trp) in all 16 species, and AUA (Ile) in *E. calophyllus*, *E. nudipes* , *E. perulatus*, and *E. quinqueflorus* revealed no bias (RSCU = 1). Furthermore, the codon UUA encoding leucine (Leu) had the largest RSCU value, followed by GCU, which encodes alanine (Ala) (Table S5b and Figure S3B).

### 3.7 Positive Selection Analysis

To identify the positively selected genes (PSGs), we performed an analysis of chloroplast genes by calculating *dN*, *dS*, and *w* for all 80 PCGs across 16 *Enkianthus* species. The results revealed that most of the PCGs had *w* values less than 1, that is, they suffered from purification selection. Using the likelihood method and a 5% *p*‐value to assess statistical significance and BEB implemented to detect positively selected codon sites, we identified 12 protein‐coding genes (*accD*, *atpA*, *ccsA*, *matK*, *ndhF*, *petB*, *petD*, *psaJ*, *rbcL*, *ycf1*, *ycf2*, and *ycf3*) under positive selection with positively selected codon sites in all tests, accounting for 15% of all tested genes (Table S6 and Figure 5A). Among these, five PSGs (*accD*, *atpA*, *matK*, *ndhF*, and *psaJ*) were exclusively detected by using branch/species‐specific LRTs, while four genes (*ccsA*, *petB*, *rbcL*, and *ycf3*) were only identified by site‐model tests for all branches, and three PSGs (*petD*, *ycf1*, and *ycf2*) were overlapped between the two tests (Figure 5A). In total, 8, 3, and 1 positively selected genes were detected in the LSC, SSC, and IR regions, respectively (Table S6a). The 12 positively selected genes were functionally classified as seven photosynthesis, three other genes, and two unknown functional genes (Table S6b). Also, the *ycf2* gene region harbored the most sites (11) under positive selection after BEB tests, followed by *ycf1* (6), *atpA* and *matK* (4), *petD* and *ycf3* (3), *petB* (2), and the other genes had only one active site (Table 3).

**TABLE 3:** Positively selected codon sites for positively selected genes in the *Enkianthus* chloroplast genomes.

Gene	−ln L	Positively selected sites
*accD*	2501.328	332K (0.963*)
*atpA*	2121.426	255R (1.000**); 391T (1.000**0); 460K (1.000**); 463V (1.000**)
*ccsA*	1394.074	95I (0.997**)
*matK*	1832.133	57R (0.955*); 64L (0.996**); 268A (0.955*); 331K (0.955*)
*ndhF*	3265.194	745L (0.984*)
*petB*	950.326	1L (1.000**); 2N (0.993**)
*petD*	715.738	1I (1.000**); 2P (1.000**); 3I (1.000**)
*psaJ*	209.251	43S (0.990*)
*rbcL*	2113.741	142V (0.951*); 145V (0.951*); 225I (0.995**); 251L (0.975*); 328A (0.999**)
*ycf1*	10891.766	690L (0.989*); 708A (1.000**); 951R (0.955*); 979F (0.979*); 1580E (0.986*); 1704Q (0.998**)
*ycf2*	9811.897	1522K (0.982*); 1569R (0.983*); 1572N (0.981*); 1577I (0.983*); 1884R (0.983*); 2018P (0.983*); 2019L (0.983*); 2144I (1.000**); 2149E (0.982*); 2161Q (0.983*); 2171R (0.951*)
*ycf3*	590.210	5R (0.950*); 109F (0.994**); 111G (0.950*)

*p < 0.05; **p < 0.01.

Lineage‐specific tests detected no PSGs in sect. *Andrommedina* and sect. *Enkiantella*. However, the two photosynthesis genes (*atpA* and *ndhF*) and/or two other genes (*matK*, *accD*) and/or one unknown functional gene (*ycf1*) were found to be under positive selection in species of sect. *Enkianthus*, including *E. calophyllus*, *E. perulatus*, *E. quinqueflorus*, *E. serotinus* , *E. serrulatus* , and *E. tubulatus* (Figure 5A). The photosynthesis gene *psaJ* was positively selected in species of sect. *Meisteria*, including *E. campanulatus* , *E. cernuus* , and *E. sikokianus* (Figure 5A). Besides these PSGs, the photosynthesis *petD* and unknown functional gene *ycf2* showed signatures of positive selection in members of both sections (Figure 5A). In addition, species‐specific tests unambiguously detected three PSGs (*matK*, *petD*, and *ycf1*; Figure 5A). Of them, *matK* and *ycf1* were under positive selection exclusively in *E. tubuatus* and *E. sikokianus*, respectively, while *petD* was positively selected in six species (*E. calophyllus*, *E. perulatus*, *E. quinqueflorus*, *E. serotinus* , *E. serrulatus* , and *E. sikokianus*; Figure 5A).

## 4 Discussion

### 4.1 Genome Size, Gene Content and Structure of *Enkianthus* Plastomes

The plastomes of *Enkianthus* show some characteristics different from other photosynthetic Ericaceae species reported previously (Table S7). For instance, the plastome sizes of *Enkianthus* (157,053–159,852 bp) are comparable to most of the published conserved plastid genomes (usually 120–160 kb, Gao et al. 2010), but are usually shorter in length than the plastid genomes of other Ericaceae species, for example, *Pyrola rotundifolia* (168,995 bp), *Vaccinium macrocarpon* (176,045 bp) and *Rhododendron delavayi* (202,169 bp). In comparison, the size of the SSC region is close to that of the IR region in *Enkianthus*, while in *Pyrola*, *Vaccinium*, and *Rhododendron*, the former is remarkably smaller than the latter. Furthermore, *Enkianthus* plastomes display higher overall GC content than other Ericaceae species. The GC content follows the typical plastome pattern: IR > LSC > SSC. This elevated GC content in IR regions may contribute to their sequence conservation through enhanced genome stability (Huang et al. 2024). Overall, the plastomes of *Enkianthus* are similar to those of closely related Ericales families (e.g., *Clethra fargesii* and *Styrax japonicus* ) in both genome size and GC content (Table S7). The genome size and GC content probably contain some phylogenetic signals; for example, the genome size of *Rhododendron* generally increases from the early‐branching to late‐branching lineages (Xia et al. 2024). The smaller plastome size and higher GC content may reflect *Enkianthus*'s earliest‐diverging position within Ericaceae (Anderberg 1994; Kron et al. 2002).

In general, the *Enkianthus* plastomes maintain conserved gene content at IR boundaries, though minor positional fluctuations were observed at IR/SC junctions. These variations primarily involve the relative positions of *ndhF* and *ycf1*, along with the adjacent genes including *rpl23*, *trnI‐CAU*, *trnN‐GUU*, *rpl32*, and *trnH‐GUG* (Figure 2). Notably, *ndhF* exhibits distinct sectional patterns—while located within the SSC region in sect. *Enkianthus* (a phylogenetically divergent clade as illustrated below), it extends into the IRb region in the other three sections. The distance of *ycf1* from the SSC/IRa junction is also seemingly related to phylogenetic relationships among the four sections of *Enkianthus*. We observed a progressive increase in this distance from the earliest‐branching lineages (84/85 bp in sect. *Enkianthus*) to more late‐diverging lineages (117 bp in sect. *Andromedina*, and 157/158 bp in sects. *Enkiantella* and *Meisteria*), suggesting this feature may serve as a phylogenetic marker for sectional delineation within the genus. Moreover, the near‐identical IR boundary structures between sect. *Enkiantella* and sect. *Meisteria* provide structural evidence supporting Zhou et al. (2024) hypothesis that sect. *Meisteria* represents the maternal progenitor of sect. *Enkiantella*.

In this study, a total of 227–247 SSRs were identified in *Enkianthus*, much more than those reported in other family of Ericales (e.g., 73 in *Clethra fargesii*, and 65 in *Actinidia chinensis* ) (Table S7). This finding aligns with previous reports suggesting that Ericaceae species have a large increase of repetitive DNA fraction in plastomes (Li et al. 2020; Shen et al. 2020), which raises a challenge for assembling them to a complete circular structure (Mo et al. 2022). In addition, we detected complicated repeats, including tandem, palindromic, and dispersed repeats. Notably, Rd. repeats displayed high variability compared to the conserved Rt and Rp repeats, suggesting their potential role as key drivers of structural evolution in *Enkianthus* chloroplast genomes.

Nucleotide diversity (π) analysis of *Enkianthus* plastomes revealed that the LSC and SSC regions exhibited comparably higher variations than the IR regions. Moreover, the non‐coding regions generally displayed greater nucleotide diversity, particularly in certain intergenic spacer regions. These findings are congruent with previous chloroplast genome studies of most angiosperms (Chi et al. 2020). The variability in *Enkianthus* was very high, with 22 hypervariable loci identified within the IGS regions. In contrast, the CDS regions were more conserved, except for *ycf1*. The elevated polymorphism in non‐coding regions enables finer discrimination among closely related species and could complement established barcoding markers (e.g., *ITS*). Consequently, these hypervariable regions represent candidate markers for species delimitation of *Enkianthus* and further phylogeographic investigations.

### 4.2 Phylogenetic Relationship and Divergence Time of *Enkianthus*

The phylogenetic analysis strongly supported the monophyly of four sections (sect. *Enkianthus*, sect. *Meisteria*, sect. *Enkiantella*, and sect. *Andromedina*), and intersectional relationships were generally consistent with recent phylogenies based on multiple cpDNA regions (Zhou et al. 2024). However, *E. tubulatus* was sister to *E. serrulatus* based on the previous cpDNA tree and our PCG dataset, but was sister to *E. serotinus* based on the WG dataset in the current study. It is noteworthy that *E. tubulatus* has been speculated to be of hybrid origin due to the conflict between nuclear and plastid phylogenies (Zhou et al. 2024). Nevertheless, the conflict between the WG and PCG datasets in this study makes it still unclear which species ( *E. serrulatus* or *E. serotinus* ) serves as the maternal progenitor of *E. tubulatus*, and denser sampling and population‐level phylogenetic analyses are needed to resolve this issue.

Our estimated age for the stem Ericaceae (~85 Mya) is slightly younger than previous estimates (~90 Mya; Rose et al. 2018), supporting a Late Cretaceous origin for the family. Within Ericaceae, *Enkianthus* represents the earliest‐diverging lineage, with its origin estimated at 68.53 Mya (95% HPD: 61.65–77.97 Mya). This timeframe coincides with the Cretaceous‐Paleogene (K‐Pg) mass extinction event (66 Mya; Jablonski and Chaloner 1994), suggesting the genus may have emerged during this critical period of biotic turnover. However, the first major divergence within *Enkianthus* occurred much later, at 6.88 Mya (95% HPD: 3.13–15.11 Mya), likely triggered by the global cooling and aridification in Central Asia during the Miocene to Pliocene transition (Zachos et al. 2001). Further divergence among sections, and also species occurred since the Pliocene onward and throughout the Quaternary glacial/interglacial cycles alongside rapid and intense climatic fluctuations, which might have provided numerous ecological niches and facilitated the diversification of the genus. Collectively, our results based on chloroplast genome suggest an origin of *Enkianthus* at the Cretaceous‐Paleocene boundary but followed by recent rapid radiation in the evolutionary history.

### 4.3 Codon Usage Bias and Genome‐Wide Scanning for Positively Selected Chloroplast Genes

Although synonymous codons encode the same amino acids, different synonymous codons may have different frequencies of use, a phenomenon termed codon usage bias (Hershberg and Petrov 2008). Analysis of codon usage patterns is key to uncovering the evolutionary characteristics of genomes and selection pressure on genes (Morton 2003). The plastomes of *Enkianthus* contain a higher number of codons (23,232–23,939) in comparison with what was observed in other Ericaceae species [e.g., *A. unedo* with 17,980 (Martínez‐Alberola et al. 2013) and *R. pulchrum* with 8693 (Shen et al. 2020)], despite its smaller size in length. Among amino acids, leucine is the most frequent amino acid, while cysteine is the least frequent—a pattern consistent across the aforementioned species. Notably, the codon usage in *Enkianthus* is biased toward high representation of A and U at the third codon position, which is congruent with other reported Ericaceae species (Table S7). These codon preferences in plastomes may reflect molecular adaptation to the unique selective pressures of the elfin forest ecosystem.

To investigate selective pressures in *Enkianthus* plastomes, we calculated the ratio of *dS* and *dN* changes at each codon and performed a series of LRT tests and BEB analyses. The results indicated most of the positively selected genes (PSGs) were functionally associated with photosynthesis, suggesting strong adaptive evolution in this critical pathway. These PSGs were classified into five functional categories: 1. photosystem I subunits (*psaJ* and *ycf3*), 2. ATP synthase subunit (*atpA*), 3. NADH dehydrogenase (*ndhF*), 4. Cytochrome *b*6/*f* complex subunits (*petB*, *petD*), and 5. RuBisCO large subunit (*rbcL*). These genes play vital roles in photosynthesis, which could allow adaptation to highly selective conditions (i.e., strong light or photo‐oxidative stress) (Jalal et al. 2015). As most *Enkianthus* species are heliophilic montane plants, these photosynthetic adaptations may have been crucial for their niche specialization. Additionally, the other functional PSGs performed significant functions in cell survival and plant development, which may also contribute to the adaptation of species in *Enkianthus* to harsh habitat environments. For example, the *accD* gene encodes the β‐carboxyl transferase subunit of Acetyl‐CoA carboxylase that is essential and required for leaf development (Kode et al. 2005). The *ccsA* gene encodes products that are required for chloroplast c‐type cytochrome biogenesis (Xie and Merchant 1996). These genes have also previously been found to be under positive selection in other species occupying extreme environments (Dong et al. 2018; Li, Zheng, et al. 2024; Li, Nie, et al. 2024), underscoring their evolutionary significance in ecological adaptation.

Strikingly, the PSGs detected by branch‐specific tests have been found exclusively in sect. *Enkianthus* and sect. *Meisteria*, suggesting their potential role in adaptive divergence between these lineages. Sect. *Enkianthus* is mainly distributed in subtropical China, while sect. *Meisteria* is endemic to Japan. Although both regions experience monsoon climates, they differ in annual temperature range, precipitation regimes, and sunshine hours. Interestingly, the two sections exhibit distinct sets of photosynthesis‐related PSGs, likely reflecting habitat‐specific adaptive evolution in response to these differing environmental conditions. However, *petD*, a gene encoding a subunit of the cytochrome *b*6/*f* complex, emerged as a key exception, exhibiting positive selection in both sections. The cytochrome *b*6/*f* complex regulates the rate‐limiting step of the photosynthetic electron transport (Chen et al. 2024), and the *petD* gene was also detected as positively selected in six *Enkianthus* species via species‐specific tests. The conservation of *petD* selection despite habitat differences suggests it may represent a core adaptation in *Enkianthus*, while section‐specific PSGs reflect more localized evolutionary responses.

The distribution of positively selected genes (PSGs) across *Enkianthus* plastomes shows that the LSC regions are more subjected to positive selection compared to the SSC and IR regions, likely reflecting their higher gene density. However, BEB analyses suggest the *ycf2* gene in the IR region contains the highest number of positively selected sites, indicating exceptionally strong selective pressure. Although *ycf2*'s function remains poorly characterized in land plants, previous studies have reported that it is either under strong positive selection or completely lost (Park et al. 2018). Additionally, this gene also has been found to play roles in the process of adaptation in diverse plant lineages (Huang et al. 2021; Li, Zheng, et al. 2024; Li, Nie, et al. 2024; Zeb et al. 2022). In short, the 12 identified PSG genes may have aided *Enkianthus* in adaptation to extreme conditions of montane elfin forests and may serve as candidate genes for further research on the mechanisms of adaptive evolution of *Enkianthus* species.

## 5 Conclusion

This study presents the first comprehensive analysis of complete chloroplast genomes across 16 *Enkianthus* species. The plastomes exhibited the typical quadripartite structure of angiosperm plastomes and were highly conserved among the genus. Five mutational hotspot regions were revealed, which may be utilized as high‐resolution DNA markers for *Enkianthus* in future phylogenetic and phylogeographic studies. Phylogenetic results fully supported the four sections of *Enkianthus*, each as a monophyletic group. Divergence time analysis suggested that *Enkianthus* originated in the Late Cretaceous and diversified rapidly after the Lat‐Miocene. Genome‐wide scanning identified 12 genes under positive selection and revealed footprints of adaptive evolution of chloroplast genes that made *Enkianthus* species adapt to special ecological habitats. However, several limitations should be considered, including the need for whole‐genome analyses to resolve cyto‐nuclear discordance (particularly evident in *E. tubulatus*), expanded population‐level sampling across the genus' range to uncover microevolutionary adaptation mechanisms, and functional validation through transcriptomic or transgenic approaches to confirm the adaptive significance of identified genes. Despite these limitations, these findings establish *Enkianthus* as a promising model for studying plant adaptation to East Asian montane ecosystems while highlighting the importance of integrating genomic and experimental approaches in future evolutionary studies of this genus.

## Author Contributions

**Shuilian Peng:** formal analysis (lead), methodology (lead), software (lead), validation (lead), visualization (lead), writing – original draft (lead). **Zhijun Zhai:** investigation (equal), methodology (equal), software (equal), validation (equal), visualization (equal), writing – original draft (equal). **Wan Hu:** formal analysis (supporting), investigation (supporting), software (supporting). **Hua Liang:** formal analysis (supporting), investigation (supporting), resources (supporting). **Yi Yang:** conceptualization (supporting), investigation (supporting), resources (supporting), supervision (supporting). **Yixuan Kou:** writing – review and editing (supporting). **Meixia Wang:** conceptualization (supporting). **Shanmei Cheng:** methodology (supporting). **Zhiyong Zhang:** conceptualization (lead), funding acquisition (lead), investigation (lead), project administration (lead), resources (lead), supervision (lead), writing – review and editing (lead). **Dengmei Fan:** conceptualization (lead), funding acquisition (lead), investigation (lead), project administration (lead), resources (lead), supervision (lead), writing – review and editing (lead).

## Conflicts of Interest

The authors declare no conflicts of interest.

## Supporting information


**Figure S1:** The detection of branch or clade‐based genes under positive selection using the Likelihood Ratio Tests (LRTs) in the *Enkianthus* phylogeny.

**Figure S2:** Visualization of sequence alignment of the 16 chloroplast genomes. VISTA‐based identity plots exhibit sequence identities between the 16 sequenced chloroplast genomes. The gray arrows represent genes and pink regions represent non‐coding sequences.

**Figure S3:** (A) The frequency of amino acids in the protein‐coding sequences of plastomes from 16 *Enkianthus* species. (B) The RSCU values of each codon in 16 *Enkianthus* plastomes.

**Table S1:** Samples for whole‐plastome sequence analyses of *Enkianthus*.

**Table S2:** List of the sequences downloaded from Genbank for divergence time estimation.

**Table S3a:** The number and types of SSRs in 16 plastomes tested in this study.
**Table S3b:** The number and types of complex repeats in 16 plastomes tested in this study.

**Table S4a:** The nucleotide diversity of CDS regions in DnaSP software.
**Table S4b:** The nucleotide diversity of IGS regions in DnaSP software.

**Table S5a:** The amino acid usage of 80 non‐duplicated plastid genes as a whole for each tested plastome.
**Table S5b:** The codon usage of 80 non‐duplicated plastid genes as a whole for each tested plastome.

**Table S6a:** Genomic locations of the positively selected genes in the plastomes tested by site model and branch‐site model for all branches.
**Table S6b:** Functional categories of the positively selected genes in the plastomes tested by site model and branch‐site model for all branches.

**Table S7:** General characteristics of previously reported Ericales plastomes.

## Data Availability

The CPG data have been submitted to the NCBI database and received GenBank accession numbers PP737346 (*E. calophyllus*), PP727293 (*E. perulatus*), PP737348 (*E. quinqueflorus* var. *ciliatoserrulatus*), PP727290 (*E. quinqueflorus*), PP727288 ( *E. serrulatus* ), PP727297 (*E. tubulatus*), PP727294 ( *E. serotinus* ), PP727296 ( *E. nudipes* ), PP727295 (*E. subsessilis*), PP737349 ( *E. ruber* ), PP727289 ( *E. chinensis* ), PP727292 ( *E. deflexus* ), PP722976 ( *E. campanulatus* ), PP737347 ( *E. cernuus* f. *rubens*), PP737350 (*E. sikokianus*), PP727291 ( *E. cernuus* ).

## References

[ece372129-bib-0001] Anderberg, A. A. 1994. “Cladistic Analysis of *Enkianthus* With Notes on the Early Diversification of the Ericaceae.” Nordic Journal of Botany 14, no. 4: 385–401.

[ece372129-bib-0002] Beier, S. , T. Thiel , T. Münch , U. Scholz , and M. Mascher . 2017. “MISA‐Web: A Web Server for Microsatellite Prediction.” Bioinformatics 33, no. 16: 2583–2585. 28398459 10.1093/bioinformatics/btx198 PMC5870701

[ece372129-bib-0003] Benson, G. 1999. “Tandem Repeats Finder: A Program to Analyze DNA Sequences.” Nucleic Acids Research 27, no. 2: 573–580. 9862982 10.1093/nar/27.2.573 PMC148217

[ece372129-bib-0004] Chen, Q. , Y. X. Xiao , Z. Y. Wu , et al. 2024. “M‐Type Thioredoxin Regulates Cytochrome b6f Complex of Photosynthesis.” Plant Physiology 194, no. 3: 1294–1298. 38051963 10.1093/plphys/kiad646

[ece372129-bib-0005] Chen, S. F. , Y. Q. Zhou , Y. R. Chen , and J. Gu . 2018. “Fastp: An Ultra‐Fast All‐In‐One FASTQ Preprocessor.” Bioinformatics 34: i884–i890. 30423086 10.1093/bioinformatics/bty560 PMC6129281

[ece372129-bib-0006] Chi, X. F. , F. Q. Zhang , Q. Dong , and S. Chen . 2020. “Insights Into Comparative Genomics, Codon Usage Bias, and Phylogenetic Relationship of Species From Biebersteiniaceae and Nitrariaceae Based on Complete Chloroplast Genomes.” Plants 9, no. 11: 1605. 33218207 10.3390/plants9111605 PMC7699153

[ece372129-bib-0007] Daniell, H. , S. X. Jin , X. G. Zhu , M. A. Gitzendanner , D. E. Soltis , and P. S. Soltis . 2021. “Green Giant—A Tiny Chloroplast Genome With Mighty Power to Produce High‐Value Proteins: History and Phylogeny.” Plant Biotechnology Journal 19, no. 3: 430–447. 33484606 10.1111/pbi.13556 PMC7955891

[ece372129-bib-0008] Darriba, D. , G. L. Taboada , R. Doallo , and D. Posada . 2012. “jModelTest 2: More Models, New Heuristics and Parallel Computing.” Nature Methods 9, no. 8: 772. 10.1038/nmeth.2109 PMC4594756 22847109

[ece372129-bib-0009] Dellaporta, S. L. , J. Wood , and J. B. Hicks . 1983. “A Plant DNA Minipreparation: Version II.” Plant Molecular Biology Reporter 1: 19–21.

[ece372129-bib-0010] Dong, W. L. , R. N. Wang , N. Y. Zhang , W. B. Fan , M. F. Fang , and Z. H. Li . 2018. “Molecular Evolution of Chloroplast Genomes of Orchid Species: Insights Into Phylogenetic Relationship and Adaptive Evolution.” International Journal of Molecular Sciences 19, no. 3: 716. 29498674 10.3390/ijms19030716 PMC5877577

[ece372129-bib-0011] Drummond, A. J. , and A. Rambaut . 2007. “BEAST: Bayesian Evolutionary Analysis by Sampling Trees.” BMC Evolutionary Biology 7: 214. 17996036 10.1186/1471-2148-7-214 PMC2247476

[ece372129-bib-0012] Fang, M. Y. , R. Z. Fang , M. Y. He , et al. 2005. “Ericaceae.” In Flora of China, edited by Z. Y. Wu and P. H. Raven , 242–517. Science Press.

[ece372129-bib-0013] Fang, R. Z. , and P. F. Stevens . 2005. “ *Enkianthus* Loureiro.” In Flora of China, edited by Z. Y. Wu and P. H. Raven , 243–245. Science Press.

[ece372129-bib-0014] Frazer, K. A. , L. Pachter , A. Poliakov , E. M. Rubin , and I. Dubchak . 2004. “VISTA: Computational Tools for Comparative Genomics.” Nucleic Acids Research 32: W273–W279. 15215394 10.1093/nar/gkh458 PMC441596

[ece372129-bib-0015] Gao, L. , Y. J. Su , and T. Wang . 2010. “Plastid Genome Sequencing, Comparative Genomics, and Phylogenomics: Current Status and Prospects.” Journal of Systematics and Evolution 48, no. 2: 77–93.

[ece372129-bib-0016] Gao, L. Z. , Y. L. Liu , D. Zhang , et al. 2019. “Evolution of *Oryza* Chloroplast Genomes Promoted Adaptation to Diverse Ecological Habitats.” Communications Biology 2: 278. 31372517 10.1038/s42003-019-0531-2 PMC6659635

[ece372129-bib-0017] Hershberg, R. , and D. A. Petrov . 2008. “Selection on Codon Bias.” Annual Review of Genetics 42: 287–299. 10.1146/annurev.genet.42.110807.091442 18983258

[ece372129-bib-0018] Huang, R. , X. N. Xie , A. M. Chen , F. Li , E. W. Tian , and Z. Chao . 2021. “The Chloroplast Genomes of Four *Bupleurum* (Apiaceae) Species Endemic to Southwestern China, a Diversity Center of the Genus, as Well as Their Evolutionary Implications and Phylogenetic Inferences.” BMC Genomics 22: 714. 34600494 10.1186/s12864-021-08008-z PMC8487540

[ece372129-bib-0019] Huang, Y. , X. J. Jin , C. Y. Zhang , P. Li , H. H. Meng , and Y. H. Zhang . 2024. “Plastome Evolution of *Engelhardia* Facilitates Phylogeny of Juglandaceae.” BMC Plant Biology 24: 634. 38971744 10.1186/s12870-024-05293-0 PMC11227234

[ece372129-bib-0020] Jablonski, D. , and W. G. Chaloner . 1994. “Extinctions in the Fossil Record.” Philosophical Transactions of the Royal Society of London. Series B, Biological Sciences 344: 11–17.

[ece372129-bib-0021] Jalal, A. , C. Schwarz , C. Schmitz‐Linneweber , O. Vallon , J. Nickelsen , and A. V. Bohne . 2015. “A Small Multifunctional Pentatricopeptide Repeat Protein in the Chloroplast of *Chlamydomonas reinhardtii* .” Molecular Plant 8, no. 3: 412–426. 25702521 10.1016/j.molp.2014.11.019

[ece372129-bib-0022] Jarvis, P. , and E. López‐Juez . 2013. “Biogenesis and Homeostasis of Chloroplasts and Other Plastids.” Nature Reviews Molecular Cell Biology 14, no. 12: 787–802. 24263360 10.1038/nrm3702

[ece372129-bib-0023] Jensen, P. E. , and D. Leister . 2014. “Chloroplast Evolution, Structure and Functions.” F1000Prime Reports 6: 40. 24991417 10.12703/P6-40 PMC4075315

[ece372129-bib-0024] Jin, J. J. , W. B. Yu , J. B. Yang , et al. 2020. “GetOrganelle: A Fast and Versatile Toolkit for Accurate De Novo Assembly of Organelle Genomes.” Genome Biology 21: 241. 32912315 10.1186/s13059-020-02154-5 PMC7488116

[ece372129-bib-0025] Katoh, K. , and D. M. Standley . 2013. “MAFFT Multiple Sequence Alignment Software Version 7: Improvements in Performance and Usability.” Molecular Biology and Evolution 30, no. 4: 772–780. 23329690 10.1093/molbev/mst010 PMC3603318

[ece372129-bib-0026] Kearse, M. , R. Moir , A. Wilson , et al. 2012. “Geneious Basic: An Integrated and Extendable Desktop Software Platform for the Organization and Analysis of Sequence Data.” Bioinformatics 28: 1647–1649. 22543367 10.1093/bioinformatics/bts199 PMC3371832

[ece372129-bib-0027] Kode, V. , E. A. Mudd , S. Iamtham , and A. Day . 2005. “The Tobacco Plastid *accD* Gene Is Essential and Is Required for Leaf Development.” Plant Journal 44: 237–244. 10.1111/j.1365-313X.2005.02533.x 16212603

[ece372129-bib-0028] Kron, K. A. , W. Judd , P. Stevens , et al. 2002. “Phylogenetic Classification of Ericaceae: Molecular and Morphological Evidence.” Botanical Review 68: 335–423.

[ece372129-bib-0029] Kron, K. A. , and W. S. Judd . 1997. “Systematics of the *Lyonia* Group (Andromedeae, Ericaceae) and the Use of Species as Terminals in Higher‐Level Cladistic Analyses.” Systematic Botany 22: 479–492.

[ece372129-bib-0030] Kurtz, S. 2010. “The Vmatch Large Scale Sequence Analysis Software—A Manual.” Current Bioinformatics 170: 391–392.

[ece372129-bib-0031] Langmead, B. , and S. L. Salzberg . 2012. “Fast Gapped‐Read Alignment With Bowtie 2.” Nature Methods 9, no. 4: 357–359. 22388286 10.1038/nmeth.1923 PMC3322381

[ece372129-bib-0032] Li, H. , Q. Q. Guo , Q. Li , and L. Yang . 2020. “Long‐Reads Reveal That *Rhododendron delavayi* Plastid Genome Contains Extensive Repeat Sequences, and Recombination Exists Among Plastid Genomes of Photosynthetic Ericaceae.” PeerJ 8: e9048. 32351791 10.7717/peerj.9048 PMC7183307

[ece372129-bib-0033] Li, H. , Q. Q. Guo , L. Xu , H. D. Gao , L. Liu , and X. Y. Zhou . 2023. “CPJSdraw: Analysis and Visualization of Junction Sites of Chloroplast Genomes.” PeerJ 11: e15326. 37193025 10.7717/peerj.15326 PMC10182761

[ece372129-bib-0034] Li, L. , Y. Hu , M. He , et al. 2021. “Comparative Chloroplast Genomes: Insights Into the Evolution of the Chloroplast Genome of *Camellia sinensis* and the Phylogeny of *Camellia* .” BMC Genomics 22: 138. 33637038 10.1186/s12864-021-07427-2 PMC7912895

[ece372129-bib-0035] Li, X. W. , Y. Yang , R. J. Henry , M. Rossetto , Y. T. Wang , and S. L. Chen . 2014. “Plant DNA Barcoding: From Gene to Genome.” Biological Reviews 90: 157–166. 24666563 10.1111/brv.12104

[ece372129-bib-0036] Li, Y. , S. S. Zheng , T. R. Wang , et al. 2024. “New Insights on the Phylogeny, Evolutionary History, and Ecological Adaptation Mechanism in Cycle‐Cup Oaks Based on Chloroplast Genomes.” Ecology and Evolution 14: e70318. 39290669 10.1002/ece3.70318 PMC11407850

[ece372129-bib-0037] Li, Y. L. , L. Y. Nie , S. W. Deng , et al. 2024. “Characterization of *Firmiana danxiaensis* Plastomes and Comparative Analysis of *Firmiana*: Insight Into Its Phylogeny and Evolution.” BMC Genomics 25: 203. 38389079 10.1186/s12864-024-10046-2 PMC10885454

[ece372129-bib-0038] Liang, H. , L. Jiang , D. H. Zhu , J. W. Zhou , D. M. Fan , and Z. Y. Zhang . 2022. “Nuclear DNA Content (2C‐Value) and Ploidy Level of *Enkianthus* Species (Ericaceae) From China.” Guihaia 42: 58–67.

[ece372129-bib-0039] Lohse, M. , O. Drechsel , and R. Bock . 2007. “OrganellarGenomeDRAW (OGDRAW): A Tool for the Easy Generation of High‐Quality Custom Graphical Maps of Plastid and Mitochondrial Genomes.” Current Genetics 52: 267–274. 17957369 10.1007/s00294-007-0161-y

[ece372129-bib-0040] Ma, H. , Y. B. Liu , D. T. Liu , et al. 2021. “Chromosome‐Level Genome Assembly and Population Genetic Analysis of a Critically Endangered *Rhododendro*n Provide Insights Into Its Conservation.” Plant Journal 107: 1533–1545. 10.1111/tpj.15399 34189793

[ece372129-bib-0041] Martin, W. , and K. V. Kowallik . 1999. “Annotated English Translation of Mereschkowsky's 1905 Paper ‘Über Natur Und Ursprung der Chromatophoren Im Pflanzenreiche’.” European Journal of Phycology 34: 287–295.

[ece372129-bib-0042] Martínez‐Alberola, F. , E. M. Del Campo , D. Lázaro‐Gimeno , et al. 2013. “Balanced Gene Losses, Duplications and Intensive Rearrangements Led to an Unusual Regularly Sized Genome in *Arbutus unedo* Chloroplasts.” PLoS One 8, no. 11: e79685. 24260278 10.1371/journal.pone.0079685 PMC3832540

[ece372129-bib-0043] Meade, J. C. , P. H. Shah , and W. B. Lushbaugh . 1997. “ *Trichomonas vaginalis*: Analysis of Codon Usage.” Experimental Parasitology 87: 73–74. 9287961 10.1006/expr.1997.4185

[ece372129-bib-0044] Mo, Z. Q. , C. N. Fu , M. S. Zhu , et al. 2022. “Resolution, Conflict and Rate Shifts: Insights From a Densely Sampled Plastome Phylogeny for *Rhododendron* (Ericaceae).” Annals of Botany 130, no. 5: 687–701. 36087101 10.1093/aob/mcac114 PMC9670778

[ece372129-bib-0045] Morton, B. R. 2003. “The Role of Context‐Dependent Mutations in Generating Compositional and Codon Usage Bias in Grass Chloroplast DNA.” Journal of Molecular Evolution 56: 616–629. 12698298 10.1007/s00239-002-2430-1

[ece372129-bib-0046] Park, I. , S. Y. Yang , W. J. Kim , P. Noh , H. O. Lee , and B. C. Moon . 2018. “The Complete Chloroplast Genomes of Six *Ipomoea* Species and Indel Marker Development for the Discrimination of Authentic *Pharbitidis* Semen (Seeds of *I. Nil* or *I. Purpurea*).” Frontiers in Plant Science 9: 965. 30026751 10.3389/fpls.2018.00965 PMC6041466

[ece372129-bib-0047] Qu, X. J. , M. J. Moore , D. Z. Li , and T. S. Yi . 2019. “PGA: A Software Package for Rapid, Accurate, and Flexible Batch Annotation of Plastomes.” Plant Methods 15: 50. 31139240 10.1186/s13007-019-0435-7 PMC6528300

[ece372129-bib-0048] Rambaut, A. , A. J. Drummond , D. Xie , G. Baele , M. A. Suchard , and E. Susko . 2018. “Posterior Summarization in Bayesian Phylogenetics Using Tracer 1.7.” Systematic Biology 67: 901–904. 29718447 10.1093/sysbio/syy032 PMC6101584

[ece372129-bib-0049] Robinson, J. T. , H. Thorvaldsdóttir , W. Winckler , et al. 2011. “Integrative Genomics Viewer.” Nature Biotechnology 29: 24–26. 10.1038/nbt.1754 PMC3346182 21221095

[ece372129-bib-0050] Ronquist, F. , M. Teslenko , P. Van Der Mark , et al. 2012. “MrBayes 3.2: Efficient Bayesian Phylogenetic Inference and Model Choice Across a Large Model Space.” Systematic Biology 61: 539–542. 22357727 10.1093/sysbio/sys029 PMC3329765

[ece372129-bib-0051] Rose, J. P. , T. J. Kleist , S. D. Löfstrand , B. T. Drew , J. Schönenberger , and K. J. Sytsma . 2018. “Phylogeny, Historical Biogeography, and Diversification of Angiosperm Order Ericales Suggest Ancient Neotropical and East Asian Connections.” Molecular Phylogenetics and Evolution 122: 59–79. 29410353 10.1016/j.ympev.2018.01.014

[ece372129-bib-0052] Rozas, J. , A. Ferrer‐Mata , J. C. Sánchez‐Delbarrio , et al. 2017. “DnaSP 6: DNA Sequence Polymorphism Analysis of Large Data Sets.” Molecular Biology and Evolution 34: 3299–3302. 29029172 10.1093/molbev/msx248

[ece372129-bib-0053] Schwery, O. , R. E. Onstein , Y. Bouchenak‐Khelladi , Y. W. Xing , R. J. Carter , and H. P. Linder . 2015. “As Old as the Mountains: The Radiations of the Ericaceae.” New Phytologist 207, no. 2: 355–367. 25530223 10.1111/nph.13234

[ece372129-bib-0054] Shen, J. S. , X. Q. Li , X. T. Zhu , X. L. Huang , and S. H. Jin . 2020. “The Complete Plastid Genome of *Rhododendron pulchrum* and Comparative Genetic Analysis of Ericaceae Species.” Forests 11: 158.

[ece372129-bib-0055] Shendure, J. , and H. Ji . 2008. “Next‐Generation DNA Sequencing.” Nature Biotechnology 26: 1135–1145. 10.1038/nbt1486 18846087

[ece372129-bib-0056] Stamatakis, A. 2014. “RAxML Version 8: A Tool for Phylogenetic Analysis and Post‐Analysis of Large Phylogenies.” Bioinformatics 30: 1312–1313. 24451623 10.1093/bioinformatics/btu033 PMC3998144

[ece372129-bib-0057] Tsutsumi, C. , and Y. Hirayama . 2012. “The Phylogeny of Japanese *Enkianthus* Species (Ericaceae).” Bulletin of the National Museum of Nature and Science Series Botany 38: 11–17.

[ece372129-bib-0058] Xia, X. M. , H. L. Du , X. D. Hu , et al. 2024. “Genomic Insights Into Adaptive Evolution of the Species‐Rich Cosmopolitan Plant Genus *Rhododendron* .” Cell Reports 43: 114745. 39298317 10.1016/j.celrep.2024.114745

[ece372129-bib-0059] Xia, X. M. , M. Q. Yang , C. L. Li , et al. 2022. “Spatiotemporal Evolution of the Global Species Diversity of *Rhododendron* .” Molecular Biology and Evolution 39: msab314. 34718707 10.1093/molbev/msab314 PMC8760938

[ece372129-bib-0060] Xie, Z. , and S. Merchant . 1996. “The Plastid‐Encoded *ccsA* Gene Is Required for Heme Attachment to Chloroplast c‐Type Cytochromes.” Journal of Biological Chemistry 271, no. 9: 4632–4639. 8617725 10.1074/jbc.271.9.4632

[ece372129-bib-0061] Yang, Z. 2005. “Bayes Empirical Bayes Inference of Amino Acid Sites Under Positive Selection.” Molecular Biology and Evolution 22, no. 4: 1107–1118. 15689528 10.1093/molbev/msi097

[ece372129-bib-0062] Yang, Z. 2007. “PAML 4: Phylogenetic Analysis by Maximum Likelihood.” Molecular Biology and Evolution 24, no. 8: 1586–1591. 17483113 10.1093/molbev/msm088

[ece372129-bib-0063] Yao, Y. H. , B. P. Zhang , and C. Zhao . 2017. “Geographical Distribution of Cripple Tree Forest and Its Importance for Forest Line in China.” Progress in Geography 36, no. 4: 491–499.

[ece372129-bib-0064] Zachos, J. , M. Pagani , L. Sloan , E. Thomas , and K. Billups . 2001. “Trends, Rhythms, and Aberrations in Global Climate 65 Ma to Present.” Science 292: 686–693. 11326091 10.1126/science.1059412

[ece372129-bib-0065] Zeb, U. , X. K. Wang , A. AzizUllah , et al. 2022. “Comparative Genome Sequence and Phylogenetic Analysis of Chloroplast for Evolutionary Relationship Among *Pinus* Species.” Saudi Journal of Biological Sciences 29, no. 3: 1618–1627. 35280541 10.1016/j.sjbs.2021.10.070 PMC8913380

[ece372129-bib-0066] Zhang, Z. R. , X. Yang , W. Y. Li , Y. Q. Peng , and J. Gao . 2022. “Comparative Chloroplast Genome Analysis of *Ficus* (Moraceae): Insight Into Adaptive Evolution and Mutational Hotspot Regions.” Frontiers in Plant Science 13: 965335. 36186045 10.3389/fpls.2022.965335 PMC9521400

[ece372129-bib-0067] Zheng, W. , L. J. Yan , K. S. Burgess , et al. 2021. “Natural Hybridization Among Three *Rhododendron* Species (Ericaceae) Revealed by Morphological and Genomic Evidence.” BMC Plant Biology 21, no. 1: 529. 34763662 10.1186/s12870-021-03312-y PMC8582147

[ece372129-bib-0068] Zhou, C. , H. Liang , W. Hu , et al. 2024. “Molecular Phylogeny of *Enkianthus* Lour. (Ericaceae) Based on Chloroplast and Nuclear DNA Sequences With an Emphasis on the Origin of Polyploid Species.” Scientia Horticulturae 328: 112960.

