# Supplementary figures and images for: Complete Chloroplast Genome of Enkianthus Lour. (Ericaceae): Comparative Analysis, Phylogenetic Relationships, Divergence History, and Adaptive Evolution
Source: Ecol Evol. 2025 Sep 19;15(9):e72129. doi: 10.1002/ece3.72129 (PMC12446880; doi:10.1002/ece3.72129)

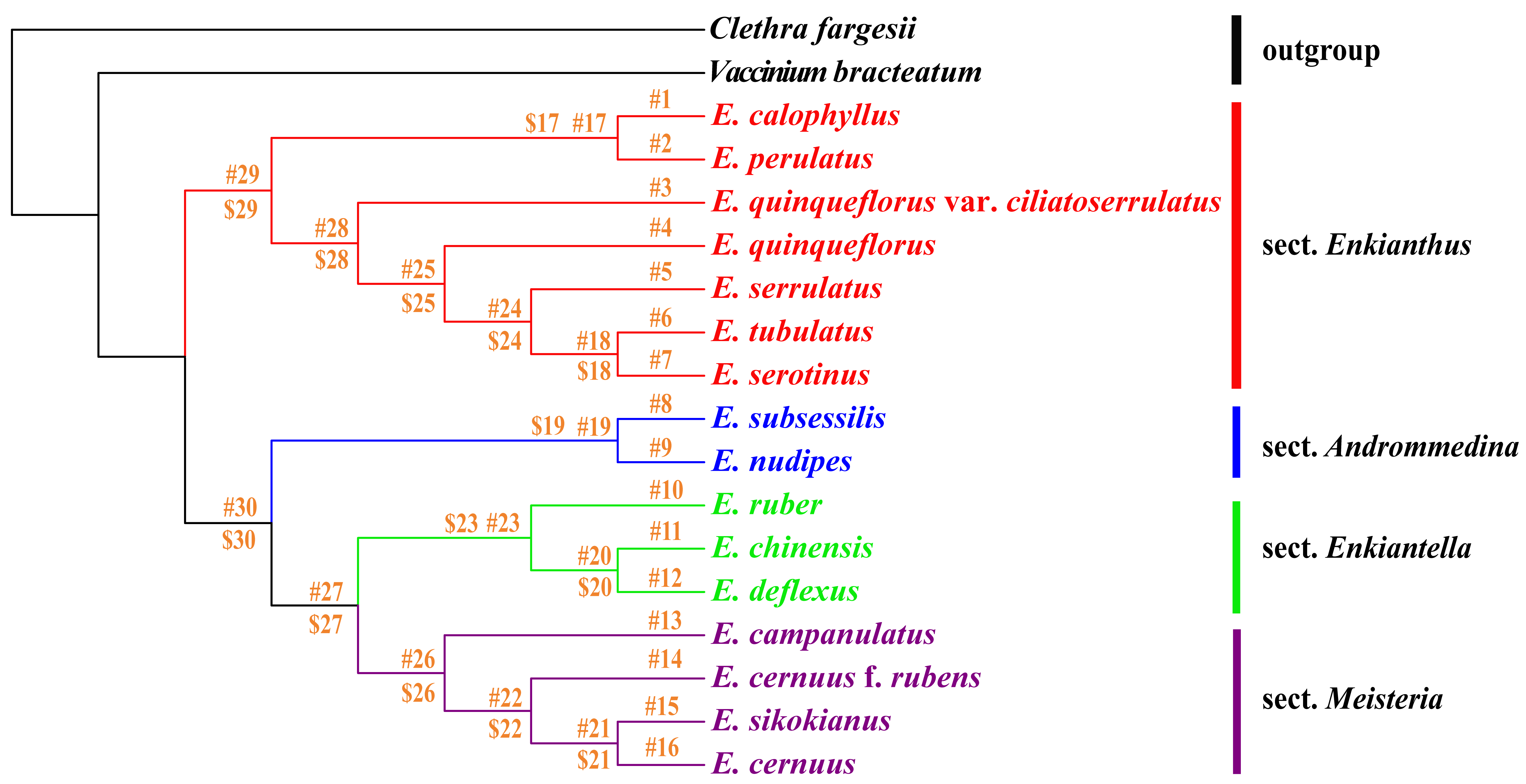

Supplement: Supplementary file 1 — Figure S1: The detection of branch or clade‐based genes under positive selection using the Likelihood Ratio Tests (LRTs) in the Enkianthus phylogeny. [file ECE3-15-e72129-s008.tif]

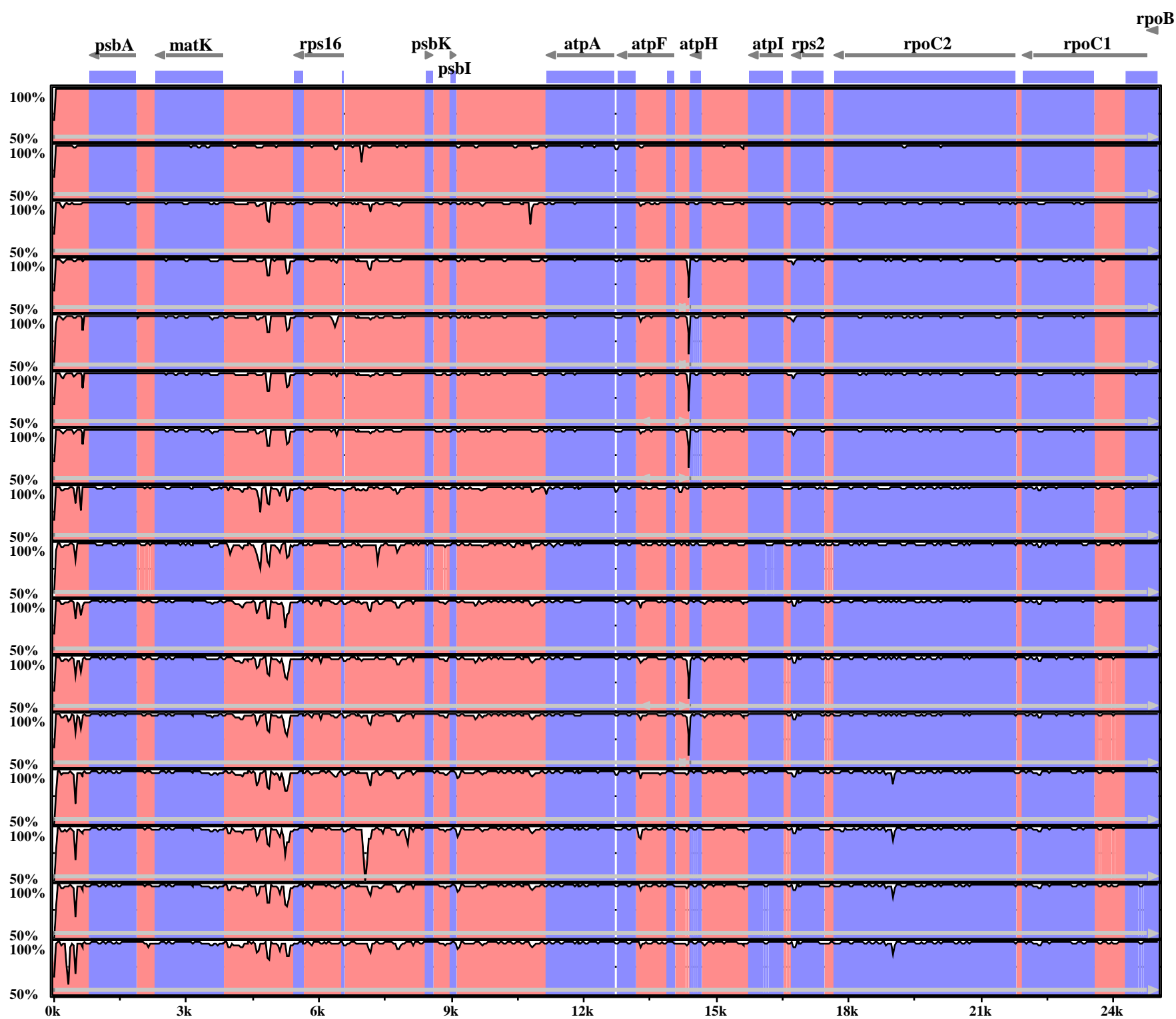

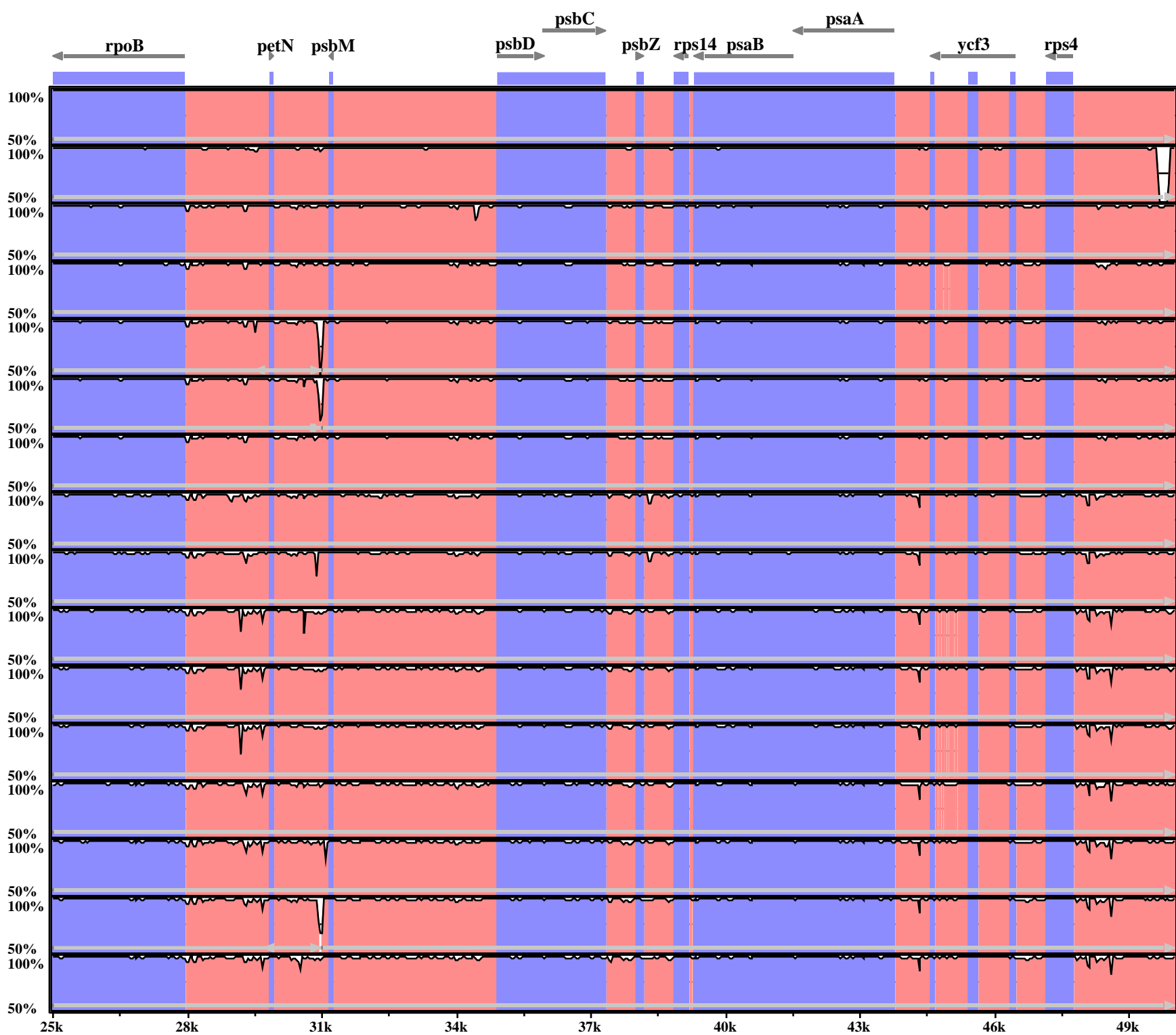

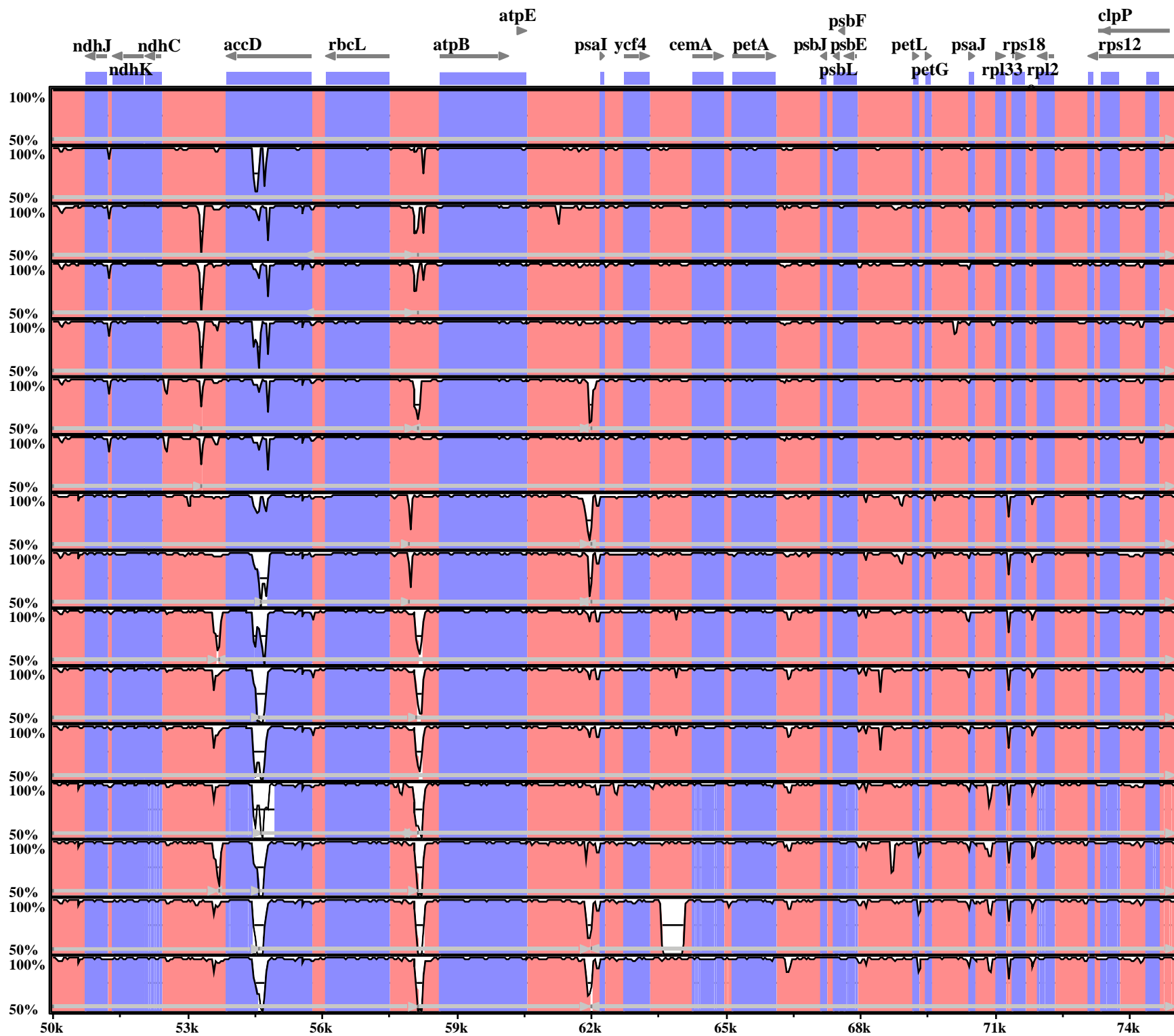

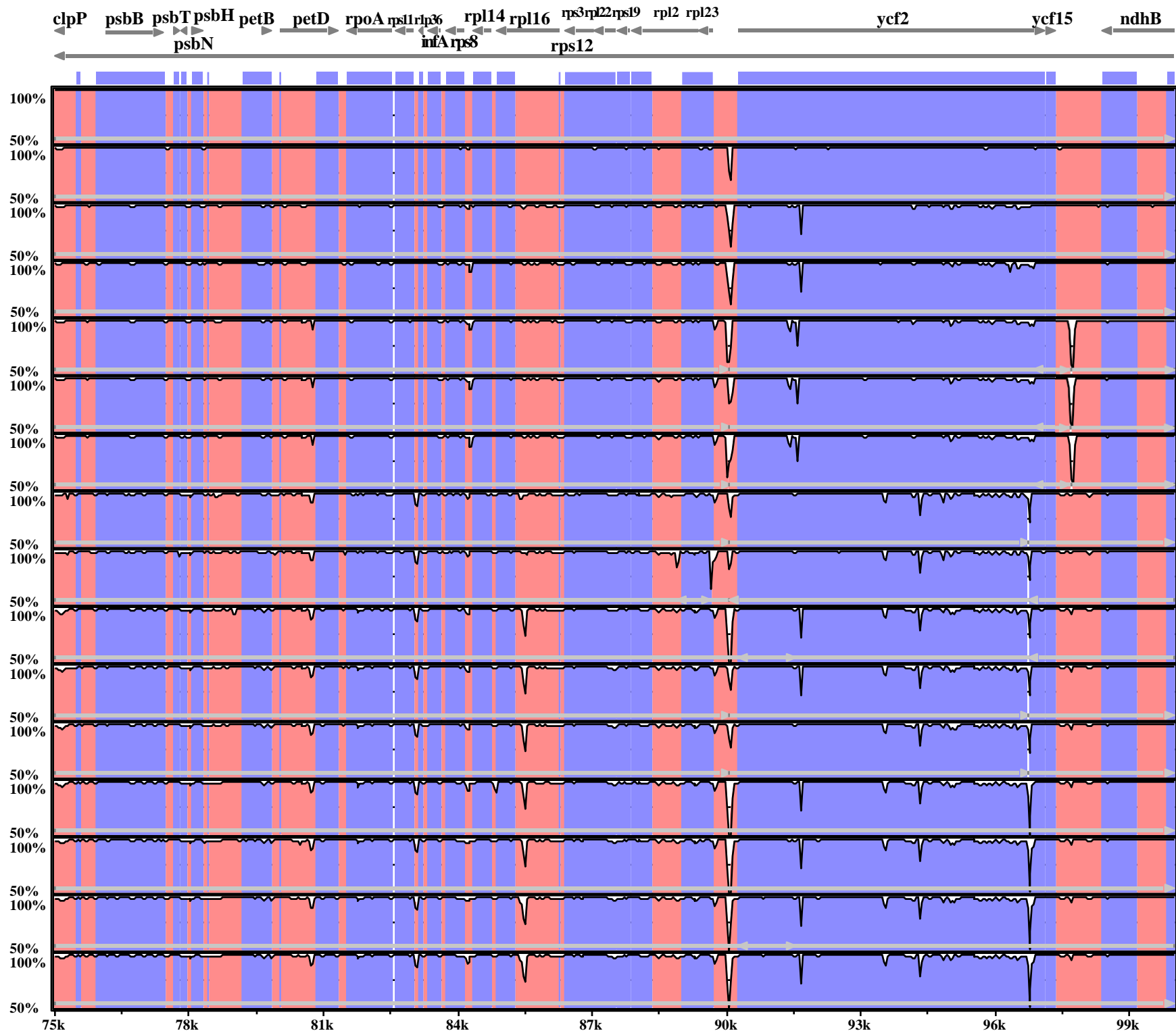

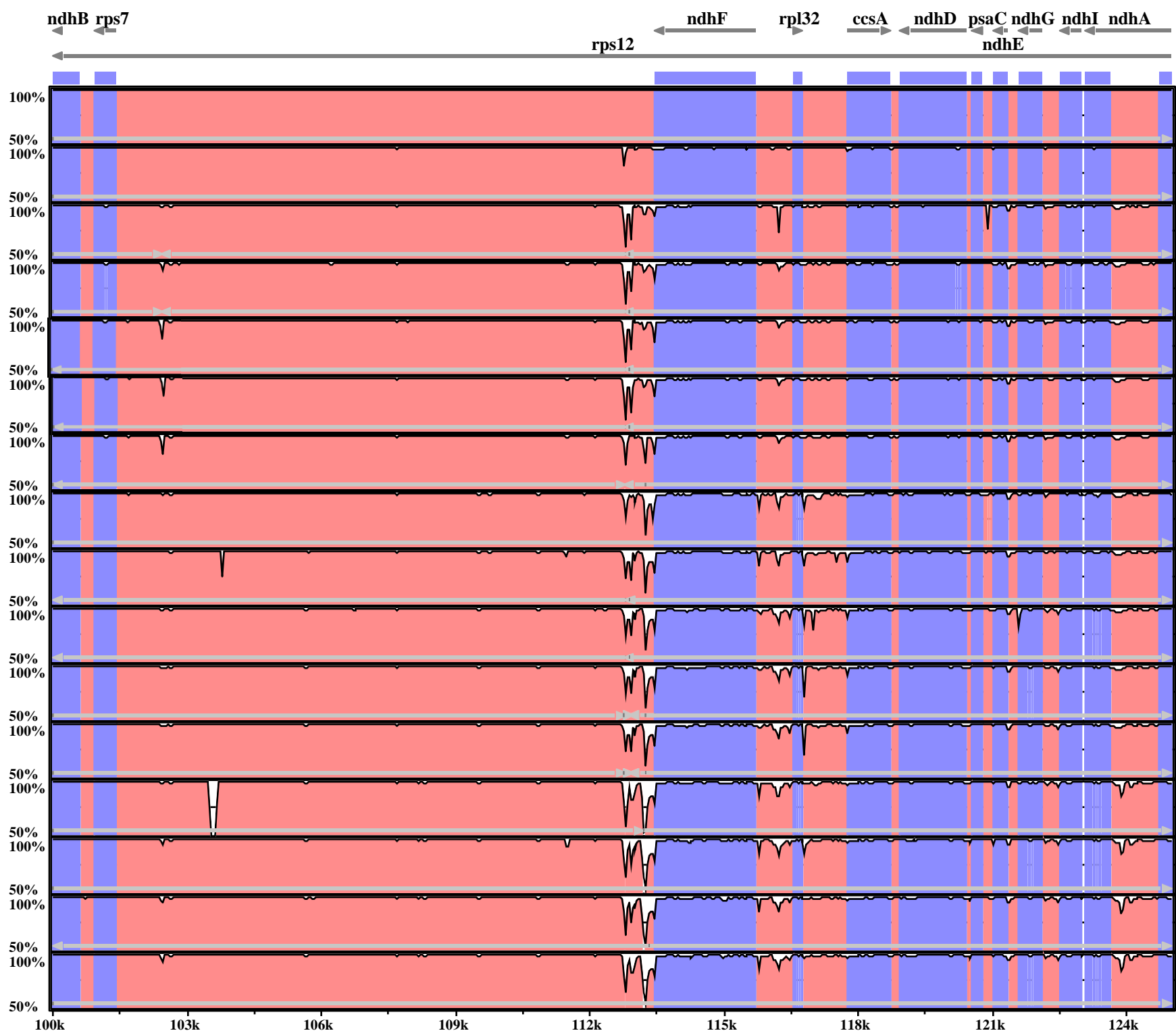

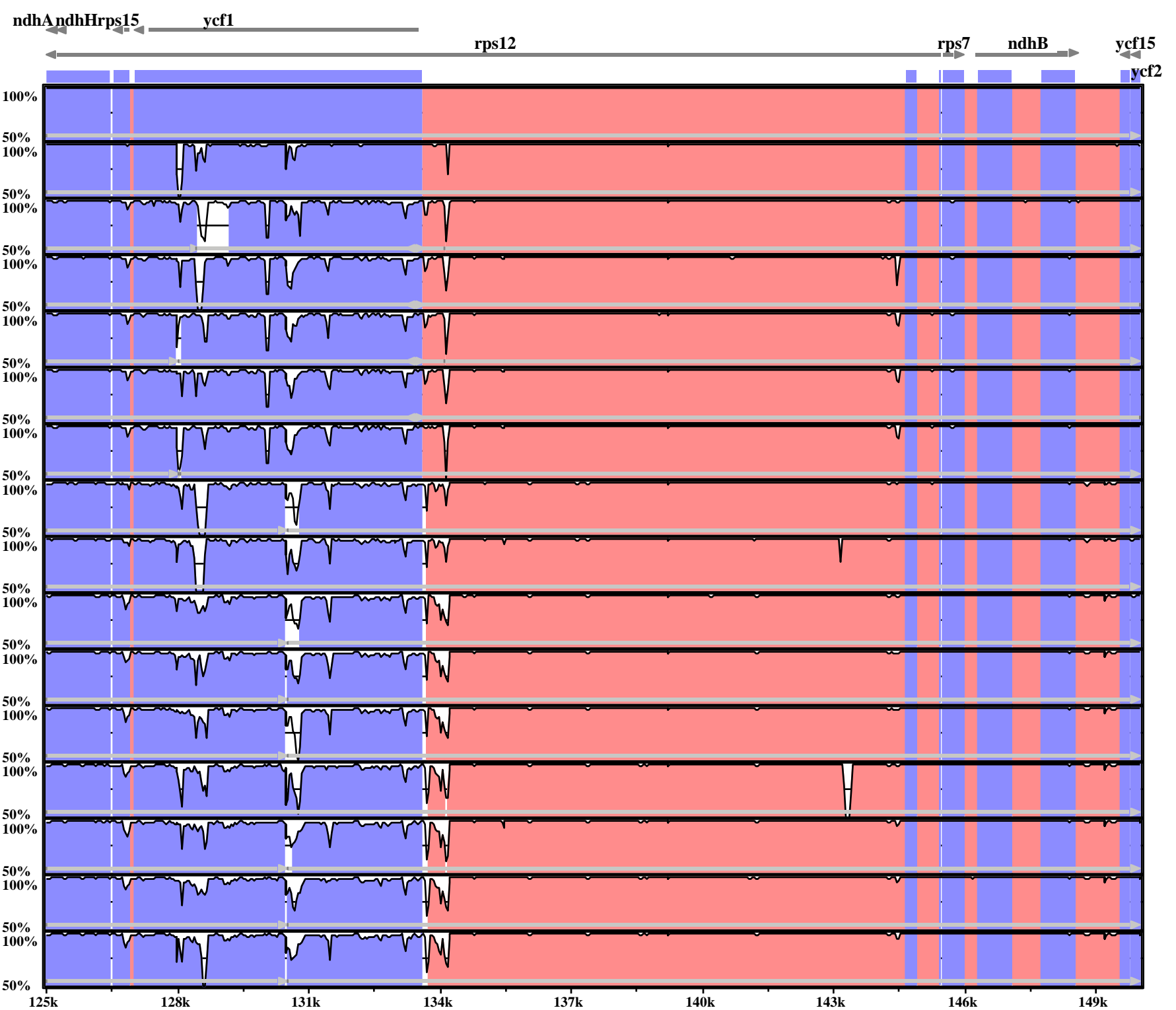

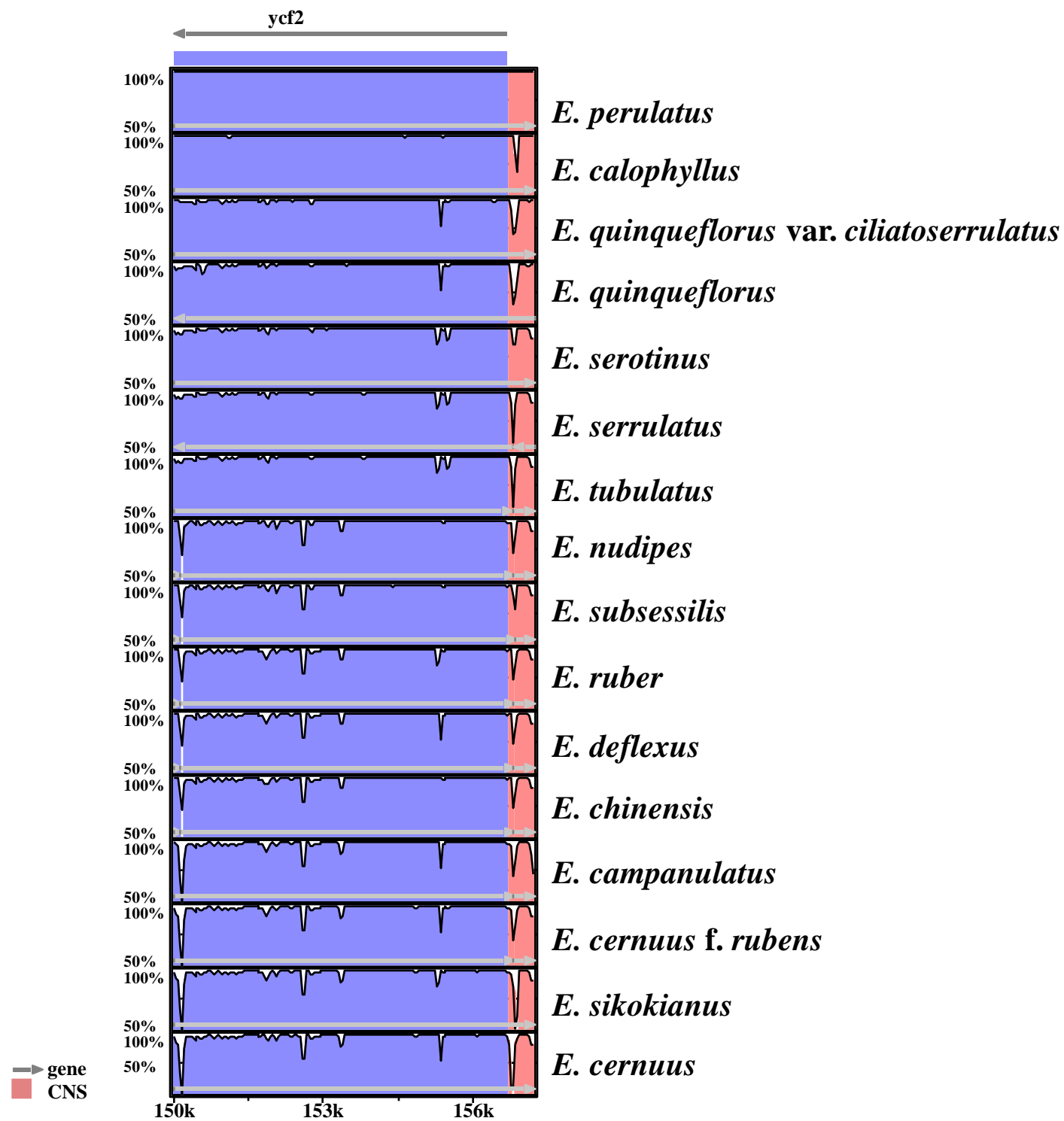

Supplement: Supplementary file 2 — Figure S2: Visualization of sequence alignment of the 16 chloroplast genomes. VISTA‐based identity plots exhibit sequence identities between the 16 sequenced chloroplast genomes. The gray arrows represent genes and pink regions represent non‐coding sequences. [file ECE3-15-e72129-s001.pdf]

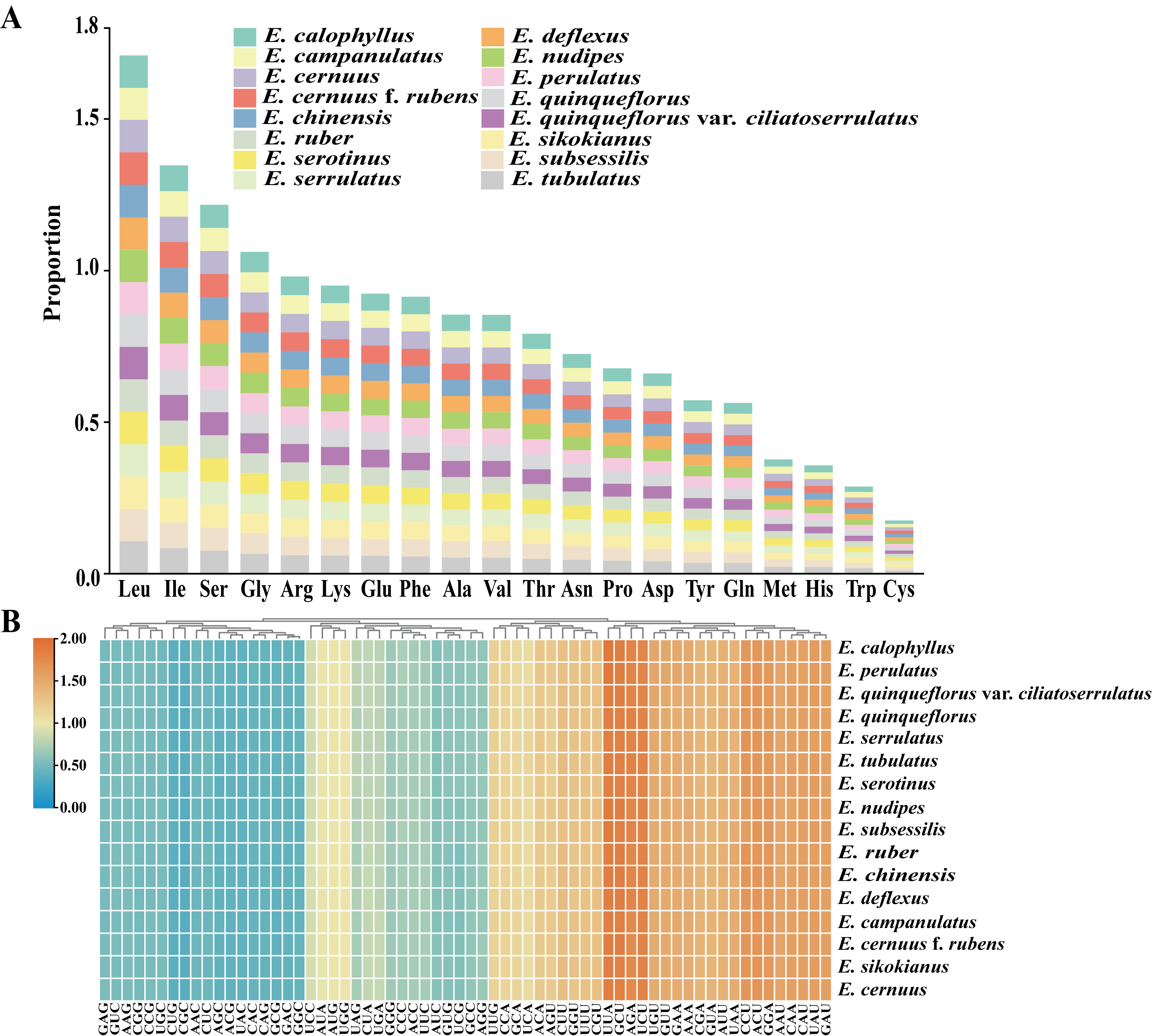

Supplement: Supplementary file 3 — Figure S3: (A) The frequency of amino acids in the protein‐coding sequences of plastomes from 16 Enkianthus species. (B) The RSCU values of each codon in 16 Enkianthus plastomes. [file ECE3-15-e72129-s004.tif]
